# Knowledge–Attitude–Practice‐Based Outdoor Exercise Education for Patients With Type 2 Diabetes: A Randomized Controlled Trial

**DOI:** 10.1155/jdr/4523789

**Published:** 2026-06-29

**Authors:** Liwen Tu, Qiang Liu, Yun Shen, Shengdi Lu, Lihua Huang, Lintao Liu

**Affiliations:** ^1^ Department of Nursing, Shanghai Geriatric Medical Center, Shanghai, China; ^2^ Department of Rehabilitation, Huashan Hospital Affiliated to Fudan University, Shanghai, China, huashan.org.cn; ^3^ Chronic Disease Epidemiology, Pennington Biomedical Research Center, Baton Rouge, Louisiana, USA, lsu.edu; ^4^ Department of Rehabilitation Medicine, Shanghai Sixth People′s Hospital Affiliated to Shanghai Jiao Tong University School of Medicine, Shanghai, China; ^5^ Department of Rehabilitation Medicine, Shanghai Jiao Tong University Affiliated Sixth People′s Hospital South Campus, Shanghai, China

**Keywords:** exercise, knowledge–attitude–practice, patient education, randomized controlled trial, Type 2 diabetes

## Abstract

**Background:**

Regular exercise is known to improve glycemic control and health outcomes in Type 2 diabetes, but many patients struggle to achieve recommended activity levels. Knowledge–Attitude–Practice (KAP)‐based education may help bridge this gap by improving exercise adherence and related outcomes.

**Objective:**

This study is aimed at assessing whether a structured KAP‐based outdoor exercise education program leads to better 6‐month glycemic and weight control than traditional exercise guidance in adults with Type 2 diabetes.

**Methods:**

We conducted a 6‐month single‐center, parallel‐group, assessor‐blinded randomized controlled trial in Shanghai, China. A total of 220 adults with Type 2 diabetes were randomized 1:1 to either a KAP‐based exercise education intervention or a traditional exercise guidance control. The KAP group received a multicomponent educational session (covering exercise knowledge, motivational counseling, and practical skills training for safe outdoor exercise) with biweekly follow‐up reinforcement, whereas the control group received one session of standard exercise advice and continued usual care. The primary outcomes (HbA1c and body weight) were assessed at baseline and 6 months. Analyses were performed on an intention‐to‐treat basis.

**Results:**

Of 242 patients screened, 220 were randomized, and a 6‐month follow‐up was completed for all. Both groups achieved significant improvements in HbA1c and modest weight loss over 6 months. At 6 months, body weight also declined by a similar amount in both groups (between‐group difference: −0.261 [95% CI −0.785, 0.262; *p* = 0.328]). HbA1c had decreased by about 1.3% in each group; the adjusted between‐group difference was −0.045% (95% CI −0.18, 0.09; *p* = 0.498). No other metabolic or functional outcomes differed significantly between groups. However, exercise adherence was higher in the KAP group: By 6 months, 80.4% of KAP participants achieved ≥ 150 min/week of exercise compared to 57.8% in the control group, and the KAP arm averaged 178.5 ± 40.3 min of exercise per week versus 154.8 ± 35.8 min in the control arm; both differences were statistically significant (*p* < 0.05).

**Conclusions:**

In this randomized trial, a KAP‐based outdoor exercise education program did not significantly outperform traditional one‐time exercise guidance in improving 6‐month glycemic control or body weight in adults with Type 2 diabetes. Both interventions yielded clinically meaningful improvements in HbA1c and other health metrics over 6 months.

**Trial Registration:**

ClinicalTrials.gov identifier: ChiCTR2500104389

## 1. Background

Type 2 diabetes mellitus (T2DM) has now reached pandemic proportions. Approximately 590 million adults worldwide are living with T2DM as of 2024, reflecting a dramatic rise from about 108 million in 1980 [1]. Pathophysiologically, T2DM is driven by insulin resistance with inadequate *β*‐cell compensation, resulting in chronic hyperglycemia that promotes both microvascular (retinopathy, nephropathy, and neuropathy) and macrovascular (coronary and cerebrovascular) complications [2]. The relationship between glycemic levels and diabetic complications is essentially continuous. Higher glycated hemoglobin (HbA1c) levels are associated with a greater risk of both microvascular and macrovascular events [3]. Given these burdens, scalable strategies that improve glycemic control and cardiometabolic risk are a public health priority.

Physical activity is a cornerstone of T2DM management. Both aerobic exercise and resistance training improve skeletal muscle glucose uptake and insulin sensitivity, and regular exercise favorably impacts weight, blood pressure, and lipid profiles [2, 4]. Meta‐analyses of randomized trials show that structured exercise training (aerobic, resistance, or combined) reduces HbA1c by 0.5%–0.7% points versus minimal advice, with larger benefits when ≥ 150 min/week are achieved [5, 6]. Consistent with this evidence base, professional guidance recommends at least 150 min per week of moderate‐intensity aerobic activity (and resistance training on 2–3 days) for most adults with diabetes, with individualized precautions for comorbidities and hypoglycemia risk [4, 7]. Nevertheless, translation into routine practice remains suboptimal: In US surveillance data, only 39% of adults with diabetes reported being physically active at recommended levels, compared with 58% of adults without diabetes [8]. This persistent gap between “knowing” and “doing” highlights the need for education that not only imparts information but also changes behavior.

The Knowledge–Attitude–Practice (KAP) model is a pragmatic framework for behavior change. In KAP‐oriented education, improving knowledge about the condition and its management is leveraged to cultivate positive attitudes (e.g., motivation, perceived benefits, and self‐efficacy), which in turn drive healthier practices (e.g., regular and safe exercise). Evidence from diabetes self‐management education (DSME) supports this pathway: A meta‐analysis demonstrated that DSME improves glycemic control at immediate follow‐up, with greater effects when contact time is higher, though benefits may wane without reinforcement, implicating attitudes and sustained practice as critical mediators [9]. Beyond meta‐analytic data, randomized trials of structured education show that theory‐informed, skills‐building programs can change behaviors and proximal outcomes. The cluster‐randomized Diabetes Education and Self‐Management for Ongoing and Newly Diagnosed (DESMOND) trial in patients with newly diagnosed T2DM evaluated a structured group education program. This intervention led to greater weight loss, higher smoking cessation rates, and more positive illness perceptions after 12 months, although it did not significantly improve HbA1c in that timeframe [10]. Similarly, a 5‐year randomized study comparing group‐based diabetes care (with ongoing education) to standard individual care found that the group approach prevented the decline in patients′ knowledge and quality of life seen with usual care. It also stabilized HbA1c and produced improvements in BMI and HDL cholesterol levels [11]. These studies suggest that education programs which explicitly target cognition, motivation, and practical skills can shift real‐world behaviors.

At the same time, large‐scale lifestyle trials underscore the central role of adherence and sustained engagement. In the Action for Health in Diabetes (Look AHEAD) trial, an intensive lifestyle intervention focused on diet and exercise counseling produced greater initial weight loss, fitness gains, and improvements in HbA1c compared to standard care. However, despite these early benefits, this intensive intervention did not lead to fewer cardiovascular events over long‐term follow‐up, likely because sustaining such behavior changes over many years proved challenging [7]. Collectively, the literature supports exercise as effective for glycemic control, while emphasizing that educational strategies must bridge the knowledge–behavior gap and be designed for adherence, reinforcement, and safety [4–7, 9–11].

Building on this evidence and aligned with our prespecified protocol, we developed a KAP‐based exercise education program that integrates targeted knowledge transfer (benefits, safety, and guidelines), attitude formation (addressing barriers, motivation, and self‐efficacy), and practice training (goal setting, intensity monitoring, and problem solving), contrasted against traditional one‐time exercise advice.

According to the KAP conceptual framework, we hypothesize that, compared with traditional exercise guidance, a KAP‐based exercise education program will produce sequential improvements along the KAP pathway: first, by enhancing exercise‐related knowledge and fostering more positive attitudes toward physical activity; second, by translating these cognitive and motivational gains into greater adherence to recommended exercise levels; and ultimately, by achieving superior improvements in the coprimary outcomes of HbA1c and body weight over 6 months. Body weight and HbA1c are designated as coprimary endpoints, while exercise adherence, KAP questionnaire scores, and other metabolic, functional, and quality‐of‐life measures are prespecified secondary outcomes. The purpose of this randomized controlled trial is to rigorously compare these two approaches on metabolic, behavioral, and safety outcomes in adults with T2DM.

## 2. Methods

### 2.1. Study Design and Setting

This study was a single‐center, prospective, parallel‐arm randomized controlled trial conducted from March 14, 2022, to March 29, 2024, at Shanghai Sixth People′s Hospital. The trial included a 6‐month intervention period with follow‐up, and outcome assessments were conducted at baseline (preintervention), 3 months, and 6 months. The design and conduct followed Consolidated Standards of Reporting Trials (CONSORT) guidelines. Ethical approval was obtained from the Institutional Review Board, and all procedures were carried out in accordance with the Declaration of Helsinki. Written informed consent was obtained from all participants prior to enrollment; patients were fully informed about the two interventions and the study′s randomized design.

### 2.2. Ethical Considerations

The study received ethical approval from the Ethics Committee of Shanghai Sixth People′s Hospital (IRB Approval No.: 2022‐KY‐041(K)) and was conducted in strict accordance with the principles outlined in the Declaration of Helsinki. All participants provided written informed consent prior to enrollment in the study. Additionally, the study protocol was officially registered with the Chinese Clinical Trial Registry (Registration Number: ChiCTR2500104389). To ensure privacy and confidentiality, all collected data were coded (deidentified) and securely stored using the hospital′s electronic data capture system on local servers, accessible only to the research team. No financial compensation or other incentives were provided to participants for their involvement in this study.

### 2.3. Participants

Eligible participants were adults with T2DM meeting predefined inclusion criteria (Table 1). Key exclusion criteria included conditions of Type 1 diabetes or other specific types of diabetes, severe diabetic complications that make exercise unsafe, and any other significant medical condition that precludes exercise (Table 1).

**Table 1:** Eligibility criteria of the study.

Inclusion criteria	Exclusion criteria
1. Adults (age ≥ 18 years) with Type 2 diabetes mellitus, diagnosed according to ADA criteria (HbA1c ≥ 6.5%, FPG ≥ 126 mg/dL, etc.) or documented in medical records 2. T2DM duration of at least 6 months, with patients on stable therapy (lifestyle ± oral hypoglycemics and/or insulin) for the past 3 months 3. Medically cleared by their physician to participate in a moderate‐intensity exercise program (no acute contraindications to exercise) 4. Able to ambulate and perform outdoor physical activity 5. Provided written informed consent to participate and willing to be randomized and followed for 6 months	1. Type 1 diabetes or other specific types of diabetes; gestational diabetes 2. Severe diabetic complications that make exercise unsafe: For example, advanced proliferative retinopathy at risk of retinal hemorrhage, unstable angina or recent cardiovascular event, uncontrolled hypertension (≥ 180/100 mmHg), end‐stage renal disease, severe peripheral neuropathy or active foot ulcer 3. Any other significant medical condition that precludes exercise (such as severe osteoarthritis, debilitating chronic obstructive pulmonary disease, uncontrolled asthma, or recent major orthopedic surgery) 4. Cognitive impairment or psychiatric illness that would interfere with understanding instructions or adherence to the intervention 5. Participation in another interventional trial or structured exercise program in the last 3 months, which could confound results 6. Pregnant or planning pregnancy in the next 6 months (due to altered exercise recommendations in pregnancy) 7. Refusal or inability to commit to follow‐up visits for 6 months

*Note:* Patients who meet all inclusion criteria and none of the exclusions will be eligible. We will also screen for exercise readiness: If needed, the Physical Activity Readiness Questionnaire (PAR‐Q) or a brief physician evaluation will be used to ensure it is safe for the individual to exercise. If any abnormal symptoms develop during the trial, medical clearance will be revisited.

### 2.4. Interventions

Participants were randomized to one of two arms: a KAP‐based exercise education intervention or a traditional exercise guidance control. Aside from the differences in exercise counseling described below, both groups continued to receive their usual diabetes care (standard medical management and routine lifestyle advice) throughout the study.

#### 2.4.1. KAP‐Based Exercise Education (KAP Group)

Participants in this group received a structured program focused on improving their exercise‐related KAP. The program was delivered by a team of certified physiotherapists (serving as exercise educators), all of whom had been trained in the intervention protocol by a senior endocrinologist. Sessions were conducted in an interactive format (typically in small groups of four to six for the knowledge and attitude components and one‐on‐one for the practical exercise training) and covered all three core components to ensure consistency. The curriculum was guided by a standardized intervention manual and accompanying educational materials. It comprised a knowledge component (60 min) covering how regular exercise benefits glycemic control and overall health, an attitude/motivation component (30 min) using a patient‐centered counseling approach grounded in motivational interviewing to help patients overcome personal barriers and set individualized exercise goals, and a practice/skills component (30 min) involving a supervised outdoor exercise session (a guided brisk walk with proper warm‐up and cooldown) in which participants practiced safe exercise techniques, learned to monitor their intensity, and followed key precautions (adequate hydration, foot care, and recognizing any hypoglycemia signs) under professional guidance. Each participant was provided with printed educational handouts summarizing the key lessons, an individualized exercise plan outline, and a logbook for recording their physical activity. In addition to the initial comprehensive education (delivered in the 1st week of enrollment), the intervention included follow‐up reinforcement: Participants received brief biweekly phone calls for the first 3 months to encourage adherence, review progress and logbook entries, and help solve any problems encountered. A booster education session was provided at the 3‐month follow‐up visit (20 min to review progress and remotivate the group), and another phone call check‐in was made around 5 months after initiation of intervention, aiming to sustain engagement through the 6‐month period. This multifaceted KAP‐based approach was designed to enhance patients′ understanding of exercise, improve their attitude and self‐efficacy, and translate that into sustained exercise practice. The detailed components of KAP‐based education are shown in the Supporting Information.

#### 2.4.2. Traditional Exercise Guidance (Control Group)

Participants in the control arm received standard exercise advice as typically given in routine diabetes care. At the baseline visit, they had a one‐time 15‐min counseling session with a physician or diabetes nurse, which reinforced the importance of regular physical activity for blood sugar control and general health. They were advised to achieve at least 150 min per week of moderate‐intensity aerobic exercise (brisk walking 30 min on most days of the week) and to incorporate simple resistance exercises a few days per week, if possible, along with general encouragement to stay active and break up sedentary time. Each control patient was provided a one‐page printed handout with an exercise plan outlining a gradual progression toward the recommended activity levels over the 6‐month period (starting with shorter, less frequent walks in the first 1–2 weeks and working up to 30‐min walks on 5 days per week by about 4 weeks, with further increases in duration or intensity as tolerated in later months). The handout also included basic safety guidelines for exercising with diabetes (such as warming up and cooling down, staying hydrated, wearing appropriate footwear, and precautions to prevent hypoglycemia or avoid exercise during periods of extreme hyperglycemia). No additional structured exercise education or scheduled follow‐up calls were provided to the control group beyond this initial session, although both groups continued to receive routine clinical care and brief encouragement about exercise at their regular clinic visits.

Participants in both groups received a structured home‐based outdoor exercise program with specified monitoring. The week‐by‐week exercise regimen for the outdoor walking program is shown in the Supporting Information, detailing the frequency and duration of walks, intensity targets, and safety guidelines for each stage of the 6‐month intervention. This plan began at a low volume and gradually increased the exercise “dosage” to reach at least the recommended 150 min per week of moderate activity by approximately Week 4, with further progression in subsequent weeks as tolerated. The regimen emphasized moderate‐intensity aerobic exercise (brisk walking), with intensity monitored by perceived exertion (participants were advised to maintain a pace at which they could talk comfortably but not sing). Each session included preparatory warm‐up exercises and postactivity cooldown stretching to prevent injury. Basic safety measures were reinforced throughout (wearing appropriate footwear, staying hydrated, and checking blood glucose when indicated, especially for those on insulin). Simple resistance exercises (such as body weight and elastic band exercises for major muscle groups) were introduced after the initial weeks and performed on 2 days per week to complement aerobic training and improve strength. Participants used personal logbooks to document each exercise session (activity type and duration), and these logs were reviewed by the physiotherapists during biweekly calls and clinic visits to monitor adherence. The exercise plan was tailored to individual capabilities: Patients with lower baseline fitness or medical limitations started at a gentler pace or shorter duration and progressed more gradually, whereas those with higher initial fitness could advance more quickly. The physiotherapists adjusted the program as needed based on each patient′s progress, gradually increasing the weekly walking volume by roughly 10% when appropriate or temporarily reducing intensity if a patient experienced fatigue, discomfort, or any safety concerns. In this way, the outdoor exercise intervention was dynamically regulated to ensure that each participant exercised safely and optimally toward their goals. Importantly, because both groups received this identical structured exercise program with monitoring, the between‐group contrast in this trial was primarily in the educational and motivational approach (i.e., the knowledge and attitude components of the KAP framework) rather than in the exercise regimen itself (the practice component). This design was adopted to ensure ethical equipoise by providing all participants with a guideline‐concordant exercise plan, while testing whether additional KAP‐based education could enhance adherence to and outcomes from that plan. All exercise sessions took place in typical urban outdoor settings. Specific environmental conditions (e.g., ambient temperature, air quality, and terrain) were not systematically monitored during these sessions. Pharmacological management of diabetes was standardized across both groups using a fasting plasma glucose (FPG)–driven medication adjustment protocol, described in detail in the Supporting Information. Study physicians, blinded to exercise‐group assignment, managed antidiabetic medications for all participants according to this algorithm throughout the 6‐month study period. The protocol followed a stepwise escalation strategy beginning with metformin, adding sulfonylureas or DPP‐4 inhibitors as needed, and initiating or titrating insulin therapy when oral agents alone were insufficient to achieve target FPG levels (4.4–7.2 mmol/L). Importantly, glucose‐lowering medications primarily intended for weight loss or cardiorenal protection (e.g., GLP‐1 receptor agonists and SGLT2 inhibitors) were excluded. All medication adjustments during the study were documented. No pharmacological weight loss therapy was provided during the trial.

### 2.5. Outcomes and Data Collection

#### 2.5.1. Primary Outcomes

The prespecified primary outcomes were the change in HbA1c and the change in body weight from baseline to 6 months. HbA1c was measured from venous blood samples at baseline, 3 months, and 6 months, providing an index of long‐term glycemic control (approximately 3‐month average glucose levels). Body weight was measured (with light clothing and no shoes) at baseline, 3 months, and 6 months on the same calibrated scale. The primary endpoints were defined as the difference in mean change in HbA1c and in body weight over 6 months between the intervention and control groups. We also calculated total weight loss, which is the change in body weight from baseline to 3‐month follow‐up and from 3‐month follow‐up to 6‐month follow‐up.

#### 2.5.2. Secondary Outcomes

A variety of secondary outcome measures were collected to assess additional metabolic, cardiovascular, behavioral, and quality‐of‐life effects of the intervention. These secondary outcomes included the following:

1. Anthropometric and vital signs: Waist circumference (measured at the mid‐abdominal level) to assess central adiposity, resting systolic/diastolic blood pressure measured with an automated sphygmomanometer after 5 min of rest, and resting heart rate. These were recorded at baseline, 3 months, and 6 months.
2. Laboratory measures: FPG and fasting insulin levels (the latter measured to allow calculation of insulin resistance via HOMA‐IR), along with a fasting lipid profile (limited to triglycerides as the lipid parameter), were measured at baseline and follow‐up visits. Changes in these parameters provided additional information on glycemic control and metabolic health.
3. Physical function: Functional exercise capacity and strength were evaluated through performance tests. A 6‐min walk test (6MWT) was used to gauge aerobic endurance (distance walked in 6 min on a flat course), and a chair stand test (30CST, number of repetitions of standing from a seated position in 30 s) was used to assess lower body strength and endurance. These tests were conducted at baseline and 6 months to see if the intervention led to improvements in physical fitness.
4. Quality of life: Health‐related quality of life was measured using the Short Form‐36 (SF‐36) questionnaire. Participants completed the SF‐36 at baseline and at 6 months, and changes in its domain scores (physical health component and mental health component) were analyzed to determine if lifestyle changes translated into improved well‐being.
5. KAP questionnaire scores: Participants′ KAPs regarding exercise were assessed using a study‐specific KAP questionnaire developed for this trial (Supporting Information 1: Table S1 and Supporting Information). Reliability analysis of the KAP questionnaire was conducted using Cronbach′s alpha. For the knowledge section, items were coded as 1 and 0, and internal consistency was assessed using the Kuder–Richardson Formula 20 (KR‐20), which is equivalent to Cronbach′s alpha for dichotomous items. For the attitude and practice domains, Cronbach′s alpha was calculated based on Likert scale responses. Values between 0.60 and 0.80 were considered to indicate acceptable internal consistency for multidimensional KAP constructs. All reliability analyses were performed using STATA. The questionnaire was administered at baseline, 3 months, and 6 months. It yielded quantitative scores for each domain (exercise knowledge, attitude toward exercise, and self‐reported exercise practice) and an overall KAP composite score. Improvement in KAP scores in the intervention group relative to controls was a key secondary outcome, reflecting the efficacy of the educational program on patient attitudes and behaviors.
6. Exercise adherence: To objectively capture behavioral change, exercise adherence was tracked. Participants in both groups maintained exercise logbooks documenting their physical activity (type of exercise, duration, and frequency). From these, the total minutes of moderate‐intensity exercise per week were calculated at 3 and 6 months. A particularly important behavioral outcome was the proportion of participants in each group who achieved the recommended ≥ 150 min per week of moderate exercise by 6 months.

Additionally, adverse events during the study were recorded. Adverse events of interest included exercise‐related injuries (musculoskeletal problems and falls) and episodes of hypoglycemia. Participants were instructed to report any hypoglycemic symptoms or low glucose readings; such episodes were documented based on self‐reports (via logbooks and phone check‐ins). These safety data were monitored throughout the trial to ensure the interventions did not pose undue risk.

### 2.6. Data Collection

Outcome measurements were taken at three time points: baseline (prior to randomization and intervention), mid‐intervention at 3 months, and end of study at 6 months (Supporting Information 2: Table S2). In addition, participants′ baseline physical activity levels were assessed via self‐report at study entry (through the exercise log and the baseline KAP questionnaire′s practice section) to characterize their preintervention exercise habits; these baseline activity data are reported in Table 2. All measurements followed standardized procedures: For example, blood samples were drawn after an overnight fast for laboratory tests, blood pressure and heart rate measurements were taken in a seated position after rest, and trained research staff administered the functional tests and questionnaires according to protocol. Outcome assessors were blinded to group allocation (see the Randomization, Allocation, and Blinding subsection) to reduce potential measurement bias. Data were recorded on case report forms and entered into a secure trial database. To maximize follow‐up, participants received reminders for study visits, and if a visit was missed, the team attempted to contact the participant to obtain essential outcome data. Primary analyses were based on the planned in‐person assessments at 3 and 6 months.

**Table 2:** Baseline function and KAP scores of both groups (intention‐to‐treat population).

ITT	Sample characteristic	KAP group (*N* = 110)	Control group (*N* = 110)	*p* value
Physical examination outcome	Body weight (kg)	91 (6.21)	90 (7.09)	0.302
	Waist circumference (cm)	87 (8.75)	86 (7.95)	0.413
	Resting systolic blood pressure (mmHg)	129 (10.45)	128 (10.57)	0.601
	Resting diastolic blood pressure (mmHg)	89 (5.46)	89 (5.64)	0.316
	Resting heart rate (bpm)	74 (9.57)	76 (9.14)	0.150
Laboratory test outcomes	HbA1c (%)	8 (0.46)	8 (0.50)	0.524
	Fasting plasma glucose (mg/dL)	6 (0.99)	6 (0.95)	0.515
	Fasting insulin (*μ*IU/mL)	7 (1.12)	7 (1.18)	0.700
	Triglycerides (mg/dL)	9 (1.75)	9 (1.82)	0.650

Functional exercise capacity	6‐min walk test distance (m)	445 (79.33)	456 (83.77)	0.329
	Chair stand test (in 30 s)	14 (2.74)	14 (2.69)	0.552

Patient‐reported outcome measures	SF‐36: Physical functioning (score)	63 (5.49)	62 (5.69)	0.630
	SF‐36: Role‐physical (score)	62 (12.56)	62 (12.52)	0.687
	SF‐36: Bodily pain (score)	63 (7.46)	64 (7.12)	0.719
	SF‐36: General health (score)	63 (5.21)	64 (5.04)	0.743
	SF‐36: Vitality (score)	63 (7.92)	64 (6.87)	0.221
	SF‐36: Social functioning (score)	60 (6.60)	60 (7.09)	0.906
	SF‐36: Role‐emotional (score)	60 (19.04)	65 (18.66)	0.031
	SF‐36: Mental health (score)	63 (5.99)	62 (6.00)	0.139
	Physical component summary (score)	63 (4.23)	63 (4.09)	0.837
	Mental component summary (score)	61 (5.38)	63 (5.59)	0.139
KAP: Knowledge domain (true, false)	Regular exercise can help lower blood sugar levels in patients with Type 2 diabetes (no. [%])			
	True	98 (89.09)	105 (95.45)	0.077
	False	12 (10.91)	5 (4.55)	
	The recommended minimum amount of moderate‐intensity exercise for adults with diabetes is 150 min per week (no. [%])			
	True	96 (87.27)	104 (94.55)	0.061
	False	14 (12.73)	6 (5.45)	
	Exercise can improve insulin sensitivity and help insulin work better in the body (no. [%])			
	True	100 (90.91)	98 (89.09)	0.653
	False	10 (9.09)	12 (10.91)	
	People with diabetes should avoid exercise if their blood glucose is extremely high (e.g., > 250 mg/dL with ketones) or very low (< 70 mg/dL) (assesses safety knowledge) (no. [%])			
	True	104 (94.55)	105 (95.45)	0.757
	False	6 (5.45)	5 (4.55)	
	Besides blood sugar control, regular physical activity can reduce the risk of heart disease and high blood pressure (no. [%])			
	True	97 (88.18)	100 (90.91)	0.509
	False	13 (11.82)	10 (9.09)	
	Being physically active can help in weight loss or maintaining a healthy weight (no. [%])			
	True	97 (88.18)	99 (90.00)	0.665
	False	13 (11.82)	11 (10.00)	
	It is necessary to do warm‐up exercises before starting your main exercise session (no. [%])			
	Yes	103 (93.64)	97 (88.18)	0.159
	No	7 (6.36)	13 (11.82)	
	Wearing comfortable shoes and inspecting your feet are important for diabetic patients when exercising (no. [%])			
	Yes	98 (89.09)	99 (90.00)	0.826
	No	12 (10.91)	11 (10.00)	
	If I exercise regularly, I might be able to reduce my diabetes medication dose (with my doctor′s guidance) (no. [%])			
	True	85 (77.27)	93 (84.55)	0.170
	False	25 (22.73)	17 (15.45)	
	Muscle‐strengthening exercises (like lifting light weights or resistance band exercises) are recommended at least two times a week for people with diabetes (no. [%])			
	True	100 (90.91)	101 (91.82)	0.810
	False	10 (9.09)	9 (8.18)	
	Skipping exercise for a week or two will not affect diabetes control (no. [%])			
	True	24 (21.82)	21 (19.09)	0.616
	False	86 (78.18)	89 (80.91)	
KAP: Attitude domain (Likert scale, 5 for the most positive attitude)	I believe that regular exercise is an important part of managing my diabetes (no. [%])			
	Strongly disagree	0 (0.00)	0 (0.00)	0.552
	Disagree	0 (0.00)	0 (0.00)	
	Neutral	18 (16.36)	16 (14.55)	
	Agree	43 (39.09)	51 (46.36)	
	Strongly agree	49 (44.55)	43 (39.09)	
	I am confident that I can exercise regularly, even if I encounter obstacles (e.g., bad weather or a busy schedule) (no. [%])			
	Strongly disagree	0 (0.00)	0 (0.00)	0.243
	Disagree	0 (0.00)	0 (0.00)	
	Neutral	11 (10.00)	18 (16.36)	
	Agree	46 (41.82)	49 (44.55)	
	Strongly agree	53 (48.18)	43 (39.09)	
	I enjoy being physically active (no. [%])			
	Strongly disagree	0 (0.00)	0 (0.00)	0.700
	Disagree	0 (0.00)	0 (0.00)	
	Neutral	24 (21.82)	21 (19.09)	
	Agree	41 (37.27)	47 (42.73)	
	Strongly agree	45 (40.91)	42 (38.18)	
	I worry that exercise might cause me to have low blood sugar or other health problems (no. [%])			
	Strongly disagree	0 (0.00)	0 (0.00)	0.021
	Disagree	0 (0.00)	0 (0.00)	
	Neutral	31 (28.18)	15 (13.64)	
	Agree	34 (30.91)	47 (42.73)	
	Strongly agree	45 (40.91)	48 (43.64)	
	I feel motivated to exercise when I think about the benefits it can bring to my health (no. [%])			
	Strongly disagree	0 (0.00)	0 (0.00)	0.643
	Disagree	0 (0.00)	0 (0.00)	
	Neutral	30 (27.27)	24 (21.82)	
	Agree	42 (38.18)	45 (40.91)	
	Strongly agree	38 (34.55)	41 (37.27)	
	Exercise is as important as taking medication for controlling my diabetes (no. [%])			
	Strongly disagree	0 (0.00)	0 (0.00)	0.398
	Disagree	0 (0.00)	0 (0.00)	
	Neutral	26 (23.64)	18 (16.36)	
	Agree	43 (39.09)	46 (41.82)	
	Strongly agree	41 (37.27)	46 (41.82)	
	I would exercise more if I had someone to do it with or a group for support (no. [%])			
	Strongly disagree	0 (0.00)	0 (0.00)	0.353
	Disagree	0 (0.00)	0 (0.00)	
	Neutral	21 (19.09)	29 (26.36)	
	Agree	44 (40.00)	36 (32.73)	
	Strongly agree	45 (40.91)	45 (40.91)	
KAP: Practice domain (frequency or yes/no)	On average, how many days per week do you engage in at least 30 min of physical activity? (Multiple choice: 0, 1–2, 3–4, 5 days or more). This will be scored (e.g., 0 = 0, 1, 2, 3, 4, 5 points for 5+ days) (no. [%])			
	0	14 (12.73)	14 (12.73)	< 0.001
	1	30 (27.27)	26 (23.64)	
	2	11 (10.00)	20 (18.18)	
	3	26 (23.64)	21 (19.09)	
	4	23 (20.91)	25 (22.73)	
	5	6 (5.45)	4 (3.64)	
	Do you perform a warm‐up before exercising and a cooldown after exercising? (No. [%])			
	Yes	43 (39.09)	35 (31.82)	0.260
	No	67 (60.91)	75 (68.18)	
	Do you monitor your blood glucose (or pay attention to how you feel) before or after exercise sessions? (No. [%])			
	Yes	79 (71.82)	69 (62.73)	0.151
	No	31 (28.18)	41 (37.27)	
	When was the last time you exercised continuously for at least 20–30 min? (Options: Today, within this week, last week, more than 2 weeks ago, cannot remember—to gauge recency of exercise habit) (no. [%])			
	Cannot remember	8 (6.90)	6 (5.26)	< 0.001
	More than 2 weeks ago	6 (5.17)	8 (7.02)	
	Last week	30 (25.86)	26 (22.81)	
	Within this week	41 (35.34)	32 (28.07)	
	Today	25 (21.55)	38 (33.33)	
	I incorporate physical activity into my daily routine (e.g., taking the stairs and walking instead of driving short distances) (Likert 1–5 from never to always) (no. [%])			
	Never	14 (12.07)	14 (12.28)	< 0.001
	Rarely	25 (21.55)	24 (21.05)	
	Sometimes	19 (16.38)	18 (15.79)	
	Often	22 (18.97)	27 (23.68)	
	Always	30 (25.86)	27 (23.68)	
	If I miss planned exercise sessions, I make an effort to resume and continue afterward (Likert 1–5 from never to always) (no. [%])			
	Never	0 (0.00)	0 (0.00)	< 0.001
	Rarely	28 (17.98)	20 (14.18)	
	Sometimes	15 (9.62)	17 (12.06)	
	Often	27 (17.31)	32 (22.70)	
	Always	40 (25.64)	41 (29.08)	
	Do you keep a record or log of your physical activity? (No. [%])			
	Yes	53 (48.18)	46 (41.82)	0.343
	No	57 (51.82)	64 (58.18)	

*Note:* Values were reported as mean (standard deviation) for physical examination outcome, laboratory test outcomes, functional exercise capacity, and patient‐reported outcome measures. Values for all KAP domains were reported as number (percentage).

### 2.7. Sample Size Calculation

The sample size for the trial was determined by a power analysis focused on detecting a meaningful difference in HbA1c between the two groups. Assuming a between‐group difference of 0.5% points in HbA1c change over 6 months, a standard deviation of approximately 1.1% for HbA1c changes, a two‐tailed *α* of 0.05, and a power (1 − *β*) of 0.80, we calculated that approximately 100 participants per group would be required. A similar effect size and variability were assumed for body weight (detecting roughly a 4.5 kg between‐group difference with a standard deviation of about 9 kg), yielding a comparable sample size requirement. To accommodate an expected attrition of up to 10% over the 6‐month follow‐up, the target enrollment was increased to 110 participants per group (220 total). This sample size was expected to provide adequate power for the primary endpoints and several key secondary outcomes, while accounting for possible dropouts.

### 2.8. Randomization, Allocation, and Blinding

After baseline assessments, participants were randomly assigned in a 1:1 ratio to the KAP education group or the traditional guidance group. The randomization sequence was generated using a computer program, employing permuted block randomization to ensure roughly equal numbers in each group throughout the enrollment period. To further ensure balance on important prognostic factors, randomization was stratified by baseline HbA1c level (< 8.0% vs. ≥ 8.0%). An independent statistician (not involved in participant recruitment and assessment) prepared the allocation sequence.

Group assignments were concealed using sequentially numbered, opaque, sealed envelopes. Each envelope contained the assignment for the next participant in the sequence. After an eligible participant completed all baseline evaluations and provided consent, the research coordinator opened the next envelope to reveal that individual′s group allocation. This method ensured that the investigators enrolling participants had no foreknowledge of the upcoming assignment, thus preventing any potential selection bias.

Given the nature of the intervention, the trial was open‐label for participants and intervention providers. Participants inevitably knew whether they received the intensive education program or just a one‐time advice session, and the staff delivering the intervention had to be aware of the group assignment. However, measures were taken to blind those involved in outcome measurement and data analysis. The clinicians and research personnel who conducted follow‐up evaluations were not involved in the intervention sessions, and they were kept completely unaware of each participant′s group assignment throughout the entire 6‐month study period. Importantly, these outcome assessors remained blinded even as the KAP group received frequent phone calls: The reinforcement phone calls in the KAP arm were handled by separate intervention staff who had no role in outcome assessments. Participants were also reminded not to disclose any details of their exercise instruction or phone contacts to the assessors to avoid unintentionally revealing their group. Laboratory technicians analyzing blood samples were blinded, as samples were identified only by coded IDs. Furthermore, the data analysts were kept blinded to group labels when performing the statistical analyses. The dataset used for analysis had the groups labeled generically, so the analysts did not know which label corresponded to the KAP intervention until after the primary analyses were completed. By implementing thorough blinded outcome assessment and analysis procedures, the study is aimed at minimizing any bias in measurement and interpretation of results.

Blinding participants or educators was not feasible given the nature of the interventions. However, both groups were provided with as equal attention as possible in terms of study contact to prevent differential dropout and reduce the risk of unmasking group assignment. In practice, the control group received the initial counseling session and scheduled follow‐up assessment visits at the same time points as the KAP group, albeit without the additional biweekly phone calls. This design ensured that neither group had substantially more face‐to‐face interaction with study personnel, thereby minimizing obvious disparities in contact frequency that could have hinted at group allocation. We believe these precautions effectively minimized any potential for unblinding due to the KAP group′s more frequent follow‐up while maintaining participant engagement in both arms.

### 2.9. Statistical Analysis

All analyses were conducted on the intention‐to‐treat (ITT) population, including every randomized participant in their assigned group regardless of adherence or dropout. Thus, each participant was analyzed in their originally allocated group using all available data at each time point, with no imputation performed for missing follow‐up visits. Additionally, we carried out a sensitivity analysis on the per‐protocol (PP) population, defined as those who completed all scheduled follow‐up assessments, to evaluate the robustness of the findings. Missing outcome data in this trial occurred only at the level of entire follow‐up visits. If a participant missed a follow‐up visit, all outcome measures from that visit were absent (no data were missing within an attended visit). Accordingly, the linear mixed effects models incorporated all available observations for each participant without any imputation of missing values. We did not conduct formal tests for data missing completely at random (such as Little′s test) given that missingness was confined to whole visits. The PP analysis, essentially a complete case analysis of participants with full follow‐up data, yielded results consistent with those of the primary ITT analysis, as detailed in Supporting Information 3: Table S3, Supporting Information 4: Table S4, and Supporting Information 5: Table S5.

Before formal analysis, we assessed the distribution of each continuous variable using the Shapiro–Wilk test. All primary and secondary outcome measures approximated a normal distribution. For continuous outcomes measured over time (such as body weight and HbA1c), a linear mixed effects model for repeated measures was applied to the primary outcomes. The model included fixed effects for treatment group (KAP vs. control), time (categorical variable for baseline, 3 months, and 6 months), and the group × time interaction, as well as a random intercept for each participant to account for within‐subject correlations over time. This approach made use of all available data at the three time points and inherently adjusted for baseline values by modeling the change from baseline within the framework. Additionally, the baseline value of the outcome was included as a covariate in the model for adjustment; this variable was also considered a covariate to adjust for potential influence. The primary contrast of interest was the group difference in outcome change from baseline to 6 months, as captured by the group × time interaction term at the final time point. From these models, we derived the estimated mean change in each group and the between‐group difference in mean change, along with 95% confidence intervals and *p* values for the group difference.

For dichotomous outcomes (such as the proportion of participants achieving the ≥ 150 min/week exercise target at 6 months), comparisons between groups were made using the Cochran–Mantel–Haenszel chi‐square test, stratifying by relevant baseline characteristics. This stratified chi‐square test provided an assessment of group differences while controlling for a baseline factor. The results for categorical outcomes are presented with relative risk or odds ratio estimates and corresponding 95% confidence intervals to convey the magnitude of differences.

A hierarchical testing strategy was utilized for secondary outcomes to account for multiple comparisons in an efficient manner. The secondary endpoints were ordered by clinical importance prior to unblinding. We proceeded to test each secondary outcome in sequence using a significance level of 0.05. If a given secondary outcome in the hierarchy did not reach statistical significance (*p* ≥ 0.05), any subsequent secondary outcomes were not considered statistically significant even if their *p* values were below 0.05. For transparency, all secondary outcome results were still reported with their point estimates and confidence intervals, but inferential focus was limited to outcomes up until the first nonsignificant result in the ordered list.

All hypothesis tests were two‐tailed. Results are presented with point estimates and 95% confidence intervals to aid interpretation of clinical significance. *p* values are provided for primary outcomes and for secondary outcomes in the hierarchical sequence up to the point of nonsignificance, as described above. Statistical analyses were performed using statistics software (R). The reporting of this trial′s methods and results adheres to CONSORT guidelines for randomized controlled trials.

## 3. Results

### 3.1. Participants

**Figure 1:**
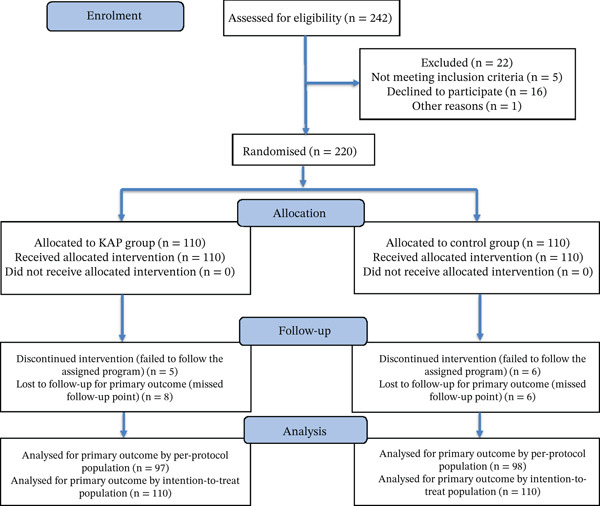
CONSORT 2025 flow diagram of the study.

Of the 242 patients assessed for eligibility, 22 were excluded (5 ineligible, 16 declined, 1 other), and 220 were randomly assigned (110 to KAP‐based exercise education and 110 to traditional exercise guidance). All randomized participants received their allocated intervention (Figure 1). During follow‐up, 13 participants (11.8%) in the KAP group and 12 (10.9%) in the control group did not complete the intervention per protocol (due to withdrawal or loss to follow‐up). Nevertheless, all 220 patients were included in the primary ITT analysis.

Baseline demographic and clinical characteristics were generally well balanced between groups. The mean age was about 47 years in the KAP group and 46 years in the control group, and roughly one‐third of participants in each group were women. Most participants were overweight, with a mean BMI of 29 kg/m^2^, and had a mean diabetes duration of about 9 years. Use of insulin and oral hypoglycemic medications was common in both arms. The only notable difference at baseline was education level, with a higher proportion of participants in the KAP group having less than a high school education compared with the control group. Baseline HbA1c was 8.0% in both groups, and physical function, blood pressure, and SF‐36 scores were comparable. Baseline KAP questionnaire results showed high exercise knowledge and positive attitudes in both arms, but poor actual exercise behavior, and only about 5% of participants were achieving the recommended 150 min per week of moderate activity (Tables 2 and 3). PP population analyses (*n* = 97 for KAP group; *n* = 98 for control group) are reported in Supporting Information 3: Table S3 and Supporting Information 4: Table S4, with baseline characteristics similar to those of the ITT population.

**Table 3:** Baseline characteristics of the KAP‐based education group and traditional education group (intention‐to‐treat population).

ITT	Characteristic	KAP group (*N* = 110)	Control group (*N* = 110)	*p* value
Baseline characteristics	Male gender (no. [%])	37 (33.6)	37 (33.6)	1.000
	Age (years)	47 (10.67)	46 (10.02)	0.725
	BMI (kg/m^2^)	29 (1.34)	29 (1.57)	0.076
	Education level (no. [%])			
	Below high school	88 (80.0)	73 (66.4)	0.022
	≥ High school	22 (20.0)	37 (33.7)	
	Insurance type (no. [%])			
	Government	73 (66.4)	63 (57.3)	0.343
	Commercial	20 (18.2)	23 (20.9)	
	Self‐financed	17 (15.5)	24 (21.8)	
	Type of work (no. [%])			
	Labor	67 (60.9)	70 (63.7)	0.676
	Nonlabor	43 (39.1)	40 (36.4)	

Medical history	Duration of diabetes (years)	9 (3.0)	9 (3.1)	0.982
	Insulin use (no. [%])	89 (80.9)	84 (76.4)	0.411
	Oral antidiabetic medications (no. [%])	92 (83.6)	83 (75.5)	0.133
	Metformin	81 (88.0)	73 (88.0)	0.938
	Sulfonylureas	22 (23.9)	19 (22.8)	0.863
	DPP‐4 inhibitors	36 (39.1)	32 (38.5)	0.887
	Thiazolidinediones	27 (29.3)	24 (29.9)	0.887
	*α*‐Glucosidase inhibitors	66 (71.7)	60 (72.3)	0.863
	Hypertension (no. [%])	8 (7.3)	12 (10.9)	0.348
	Cardiovascular diseases (no. [%])	10 (9.1)	12 (10.9)	0.653
	Atrial fibrillation (no. [%])	3 (2.7)	3 (2.7)	1.000
	COPD (no. [%])	2 (1.8)	4 (3.6)	0.408
	Arthritis/connective tissue disease (no. [%])	11 (10.0)	13 (11.8)	0.665
	Dyslipidemia (no. [%])	9 (8.2)	9 (8.2)	1.000
	Hypothyroidism (no. [%])	3 (2.7)	3 (2.7)	1.000

*Note:* Values were reported as mean (standard deviation) for age and BMI; others were reported as number (percentage).

Abbreviations: BMI, body mass index; COPD, chronic obstructive pulmonary disease; ITT, intention‐to‐treat population; KAP, knowledge, attitude, and practice.

**Figure 2:**
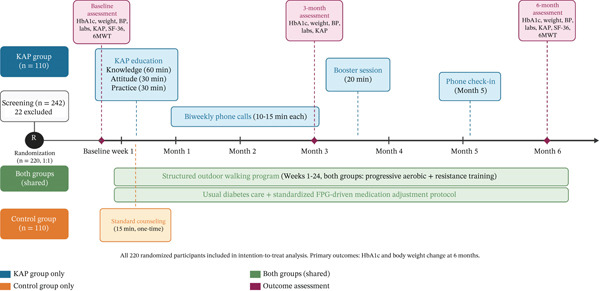
Schematic timeline of the study design. The figure illustrates the key study procedures for both the KAP‐based exercise education group and the traditional exercise guidance (control) group across the 6‐month study period. Shown are enrollment and randomization, the initial education/counseling sessions (Week 1), the shared structured outdoor walking program (Weeks 1–24), biweekly reinforcement phone calls for the KAP group (Months 1–3), a booster education session at Month 3, phone call check‐in at Month 5, and outcome assessment time points at baseline, 3 months, and 6 months.

A schematic overview of the study timeline, including intervention sessions, follow‐up contacts, and assessment time points for both study arms, is presented in Figure 2.

### 3.2. Primary and Secondary Outcomes

Both groups achieved comparable improvements in the coprimary outcomes over 6 months. Body weight decreased by approximately 3.7 kg (roughly 4% of baseline weight) in each group, with no significant between‐group difference (adjusted difference: −0.261 kg, 95% CI −0.785 to 0.262; *p* = 0.328). HbA1c decreased by approximately 1.3% points in both groups, with a negligible adjusted between‐group difference (−0.045%, 95% CI −0.18 to 0.09; *p* = 0.498). Waist circumference, blood pressure, fasting glucose, fasting insulin, and triglyceride levels all improved similarly in both arms, with no significant between‐group differences for any secondary metabolic outcome (Tables 4 and 5). No statistically significant between‐group differences were observed for any of the primary or secondary metabolic outcomes at 6 months (Tables 4 and 5). The mean weight loss corresponded to approximately 4% of baseline body weight in both groups, and neither arm attained > 5% weight loss by 6 months, underscoring the comparable and modest impact of the two interventions on body weight (Tables 4 and 5).

**Table 4:** Unadjusted changes in outcomes for the KAP‐based education and traditional education groups at Months 3 and 6 after the surgery (intention‐to‐treat population).

Intention‐to‐treat population	3 months			6 months		
	KAP group (*N* = 110)	Control group (*N* = 110)	*p* value	KAP group (*N* = 110)	Control group (*N* = 110)	*p* value
Physical examination outcome						
Body weight (kg)	89.81 (6.13)	89.02 (6.84)	0.389	86.57 (5.92)	86.03 (6.70)	0.542
Total weight loss (kg)	0.56 (0.31)	0.60 (0.33)	0.456	3.67 (0.74)	3.67 (0.87)	0.996
BMI (kg/m^2^)	−0.20 (0.05)	−0.20 (0.05)	0.882	−1.20 (0.20)	−1.19 (0.23)	0.633
Waist circumference (cm)	−3.06 (0.57)	−3.01 (0.51)	0.507	−6.09 (1.21)	−5.97 (1.31)	0.475
Resting systolic blood pressure (mmHg)	−3.10 (0.53)	−2.98 (0.58)	0.130	−6.05 (0.81)	−6.01 (0.83)	0.724
Resting diastolic blood pressure (mmHg)	−1.99 (0.55)	−2.01 (0.47)	0.773	−3.96 (0.72)	−4.00 (0.65)	0.716
Resting heart rate (bpm)	−0.99 (0.38)	−1.07 (0.46)	0.170	−2.53 (0.64)	−2.46 (0.63)	0.392

Laboratory test outcomes						
HbA1c (%)	−0.75 (0.17)	−0.76 (0.16)	0.751	−1.28 (0.18)	−1.26 (0.19)	0.409
Fasting plasma glucose (mg/dL)	−0.34 (0.08)	−0.34 (0.06)	0.706	−0.50 (0.08)	−0.50 (0.07)	0.999
Fasting insulin (*μ*IU/mL)	−0.50 (0.10)	−0.51 (0.10)	0.488	−0.99 (0.15)	−1.01 (0.13)	0.307
Triglycerides (mg/dL)	−0.00 (0.00)	−0.01 (0.00)	0.583	−0.01 (0.00)	−0.01 (0.00)	0.161

Functional exercise capacity						
6‐min walk test distance (m)	30.13 (4.18)	30.05 (4.33)	0.894	50.34 (5.38)	50.03 (5.56)	0.681
Chair stand test (in 30 s)	1.93 (0.41)	2.03 (0.46)	0.105	2.92 (0.54)	3.09 (0.50)	0.024

Patient‐reported outcome measures						
SF‐36: Physical functioning	5.45 (4.25)	5.39 (4.38)	0.930	12.60 (5.01)	13.17 (5.26)	0.423
SF‐36: Role‐physical	13.61 (12.51)	11.03 (12.47)	0.142	22.55 (15.54)	23.08 (15.46)	0.807
SF‐36: Bodily pain	6.26 (6.24)	6.03 (6.19)	0.794	12.57 (9.88)	12.76 (9.14)	0.886
SF‐36: General health	5.64 (4.16)	4.80 (4.22)	0.155	13.33 (4.79)	12.64 (4.82)	0.305
SF‐36: Vitality	4.06 (3.79)	5.78 (3.71)	0.001	11.67(4.74)	13.61 (4.26)	0.002
SF‐36: Social functioning	5.15 (5.55)	5.86 (5.56)	0.361	10.29 (8.10)	10.31 (8.21)	0.990
SF‐36: Role‐emotional	16.51 (16.77)	10.41 (20.98)	0.023	25.78 (19.32)	20.11 (19.90)	0.039
SF‐36: Mental health	4.08 (3.15)	3.76 (3.27)	0.486	8.27 (4.11)	7.50 (4.82)	0.216

KAP scores						
Scores in the knowledge domain	0.17 (0.88)	−0.05 (0.85)	0.075	0.23 (0.93)	−0.05 (0.95)	0.038
Scores in the attitude domain	2.29 (1.73)	2.27 (1.58)	0.957	2.75 (1.73)	3.01 (1.77)	0.280
Scores in the practice domain	5.65 (2.51)	5.70 (2.55)	0.905	6.80 (3.24)	6.62 (3.13)	0.672

*Note:* Values represent the mean change from baseline for each group, reported as mean (standard deviation), with *p* values comparing between‐group differences at each follow‐up point. All outcome measures were unadjusted.

Abbreviations: BMI, body mass index; KAP, knowledge, attitude, and practice.

**Table 5:** Adjusted effectiveness estimates from linear mixed effects models (intention‐to‐treat population).

Intention‐to‐treat population	3 months			6 months		
	Coefficient	95% CI	*p* value	Coefficient	95% CI	*p* value
Physical examination outcome						
Body weight (kg)	−0.268	(−1.420, 0.883)	0.648	−0.261	(−0.785, 0.262)	0.328
Total weight loss (kg)	−0.015	(−0.052, 0.023)	0.435	−0.016	(−0.106, 0.074)	0.721
BMI (kg/m^2^)	0.000	(−0.01, 0.01)	0.963	−0.001	(−0.03, 0.03)	0.930
Waist circumference (cm)	0.742	(−1.43, 2.91)	0.503	0.720	(−1.46, 2.90)	0.518
Resting systolic blood pressure (mmHg)	−0.077	(−2.71, 2.55)	0.954	−0.053	(−2.68, 2.58)	0.968
Resting diastolic blood pressure (mmHg)	0.235	(−1.05, 1.52)	0.721	0.282	(−1.01, 1.58)	0.669
Resting heart rate (bpm)	−1.931	(−4.44, 0.58)	0.132	−1.955	(−4.48, 0.57)	0.128
Laboratory test outcomes						
HbA1c (%)	−0.041	(−0.17, 0.09)	0.527	−0.045	(−0.18, 0.09)	0.498
Fasting plasma glucose (mg/dL)	−0.050	(−0.244, 0.144)	0.615	−0.045	(−0.237, 0.147)	0.644
Fasting insulin (*μ*IU/mL)	−0.074	(−0.24, 0.09)	0.367	−0.064	(−0.23, 0.10)	0.446
Triglycerides (mg/dL)	0.271	(−0.238, 0.779)	0.297	0.294	(−0.215, 0.803)	0.258
Functional exercise capacity						
6‐min walk test distance (m)	−12.662	(−34.59, 9.27)	0.258	−12.959	(−34.83, 8.91)	0.245
Chair stand test (in 30 s)	0.073	(−0.59, 0.73)	0.828	0.038	(−0.62, 0.70)	0.910
Patient‐reported outcome measures						
SF‐36: Physical functioning	0.470	(−1.17, 2.11)	0.575	0.357	(−1.39, 2.10)	0.689
SF‐36: Role‐physical	2.635	(−1.00, 6.27)	0.155	2.170	(−1.48, 5.82)	0.244
SF‐36: Bodily pain	−0.038	(−2.22, 2.14)	0.973	−0.003	(−2.40, 2.39)	0.998
SF‐36: General health	−0.224	(−1.74, 1.29)	0.772	−0.178	(−1.79, 1.43)	0.829
SF‐36: Vitality	−1.765	(−3.92, 0.39)	0.109	−2.151	(−4.37, 0.07)	0.058
SF‐36: Social functioning	−0.404	(−2.20, 1.40)	0.660	−0.284	(−2.26, 1.69)	0.778
SF‐36: Role‐emotional	−2.968	(−7.78, 1.84)	0.227	−2.183	(−6.87, 2.51)	0.362
SF‐36: Mental health	1.403	(−0.29, 3.09)	0.103	1.710	(−0.04, 3.46)	0.055
Physical component summary (score)	0.687	(−0.57, 1.95)	0.286	0.629	(−0.69, 1.95)	0.351
Mental component summary (score)	−0.947	(−2.42, 0.53)	0.208	−0.769	(−2.25, 0.71)	0.309
KAP scores						
Scores in the knowledge domain	−0.160	(−0.38, 0.07)	0.164	−0.345	(−0.60, −0.09)	0.007
Scores in the attitude domain	0.230	(−0.18, 0.64)	0.273	−0.386	(−0.91, 0.14)	0.151
Scores in the practice domain	−0.229	(−1.03, 0.57)	0.572	−0.568	(−1.26, 0.13)	0.109

*Note:* Each coefficient represents the estimated between‐group difference in the change from baseline (intervention group minus control group) for the specified outcome at that follow‐up time point. Positive coefficients indicate higher scores in the intervention group compared to the control group, whereas negative values indicate lower scores in the intervention group. Each estimate is presented with its 95% confidence interval (CI) and corresponding *p* value. All outcome measures were adjusted for baseline values in the model.

Abbreviations: BMI, body mass index; KAP, knowledge, attitude, and practice.

Functional exercise capacity improved in both groups over 6 months. The 6MWT distance and 30‐s chair stand test results are detailed in Tables 4 and 5. No clinically meaningful between‐group differences were observed for functional outcomes in the adjusted analysis. PP findings were consistent (Supporting Information 5: Table S5 and Supporting Information 6: Table S6).

Health‐related quality of life, as measured by the SF‐36, improved in both groups across most domains. Physical functioning, role‐physical, bodily pain, general health, social functioning, and mental health scores all increased from baseline, and the magnitude of improvement was similar between groups, with no significant between‐group differences for these domains.

Two SF‐36 domains showed statistically significant group differences. Vitality improved more in the control group than in the KAP group at 6 months (mean increase 13.61 vs. 11.67 points; *p* = 0.002). In contrast, the role‐emotional domain improved more in the KAP group (mean increase 25.78 vs. 20.11 points; *p* = 0.039). Thus, the control intervention was associated with greater gains in perceived energy and fatigue, whereas the KAP program yielded larger improvements in role functioning related to emotional problems (Tables 4 and 5).

The KAP‐based education had its clearest effect on exercise‐related knowledge. By 6 months, the knowledge score in the KAP group had increased by 0.23 points on the 10‐point scale, whereas the control group showed essentially no change, resulting in a significant between‐group difference (*p* = 0.038). Attitude scores improved in both arms, with similar changes and no significant differences between groups. Practice scores, reflecting self‐reported exercise behavior, also increased substantially and to a similar extent in both groups, with mean increases of about 6.6–6.8 points at 6 months (KAP 6.80 vs. control 6.62; *p* = 0.672) (Tables 4 and 5).

Reliability analysis of the KAP questionnaire using Cronbach′s alpha shows 0.77 for the knowledge domain, 0.74 for the attitude domain, and 0.72 for the practice domain, indicating acceptable internal consistency for multidimensional KAP constructs.

### 3.3. Medication Adjustments During Follow‐Up

Under the standardized FPG‐driven medication adjustment protocol, antidiabetic medication changes were common and balanced between groups during the 6‐month follow‐up. Overall, 67 participants (60.9%) in the KAP group and 64 (58.2%) in the control group had at least one medication adjustment during the study period (*p* = 0.676). The most frequent adjustments involved insulin dose titration: Among participants already receiving insulin at baseline, 61 of 89 (68.5%) in the KAP group and 55 of 84 (65.5%) in the control group underwent at least one insulin dose increase during follow‐up. Addition of a new oral antidiabetic agent occurred in 24 participants (21.8%) in the KAP group and 22 (20.0%) in the control group. Insulin therapy was newly initiated in 8 of the 21 insulin‐naïve KAP participants (38.1%) and 10 of the 26 insulin‐naïve control participants (38.5%). No significant between‐group differences were detected in any category of medication adjustment (all *p* > 0.5). These data confirm that pharmacological intensification was applied uniformly across both study arms and that the observed reductions in HbA1c (> 1% point in both groups) reflect the combined effects of increased physical activity and concurrent medication optimization, rather than exercise alone.

### 3.4. Adherence and Adverse Events

All participants had consistent access to safe outdoor walking areas throughout the study period, and none reported difficulties attributable to environmental factors that impacted their exercise adherence. Adherence to the prescribed exercise program was high in both groups, with a mild to modest advantage in the KAP group. According to the weekly exercise logbooks, participants in the KAP group reported a mean of about 123.5 ± 32.5 min per week of moderate‐intensity exercise at 3 months, compared to approximately 119.6 ± 25.7 min per week in the control group (difference not statistically significant). By 6 months, weekly exercise duration increased in both groups: The KAP group reached an average of roughly 178.5 ± 40.3 min per week, whereas the control group averaged about 154.8 ± 35.8 min per week. This represented a modest between‐group difference favoring the KAP intervention, which became statistically significant by 6 months (*p* < 0.05). Notably, a greater proportion of participants in the KAP group achieved the recommended ≥ 150 min per week of moderate exercise by the 6‐month follow‐up (80.4% in KAP vs. 57.8% in the control group), indicating better adherence in the KAP‐based education group.

Adverse events related to exercise were rare in both groups, with no serious events reported. Minor musculoskeletal complaints (transient joint or muscle pain) were noted in only two participants (5%) in the KAP group and three participants (7%) in the control group; none of these minor injuries required medical attention or led to discontinuation of the exercise program. Falls during exercise sessions were exceedingly uncommon: Only one participant (in the control group) experienced a minor fall, which did not result in any injury. Mild hypoglycemia episodes were reported in one participant from each group, all of which were self‐managed and did not require hospitalization or intervention. Importantly, no participants withdrew from the study due to exercise‐related problems, and there were no severe adverse events in either group, underscoring that the exercise interventions were safe and well‐tolerated.

## 4. Discussion

In this randomized controlled trial, both the KAP‐based exercise education program and the traditional exercise guidance approach led to improvements in glycemic control, body weight, and other metabolic outcomes during the 6‐month follow‐up. Participants in both groups also showed favorable changes in cardiovascular risk factors, functional performance, and quality‐of‐life measures over time. Notably, both groups also showed substantial gains in health‐related quality of life: SF‐36 domain scores increased by roughly 10–20 points over 6 months, exceeding the minimal clinically important difference of 5 points on the SF‐36 [12]. The intervention group demonstrated clearer gains in exercise‐related knowledge compared with the control group, while changes in attitudes and practices were similar between groups.

Despite the additional structured education and reinforcement provided in the KAP program, the overall clinical outcomes at 6 months did not differ substantially from those achieved with standard exercise advice. Both groups showed robust improvements, so there was a lack of any incremental effect from the KAP intervention. We now articulate several factors that likely explain this outcome. First, the control group was not a minimal‐care arm; it received a structured exercise counseling session and plan, which led to significant lifestyle improvements and narrowed the gap between groups. Indeed, both groups markedly increased their physical activity, a level known to significantly improve glycemic control in Type 2 diabetes. Consistent with this, HbA1c dropped by 1.3% in both groups, suggesting that basic exercise guidance alone was highly effective. Second, the 6‐month follow‐up may have been too short to capture modest longer term advantages of the KAP approach; a longer duration might be required for differences in clinical outcomes to emerge. Third, participant characteristics could have blunted the relative impact of KAP. Baseline exercise knowledge was high in both groups (a ceiling effect), and our cohort was on average younger and relatively well‐informed and motivated. Such factors would make it easy for the control arm to improve with minimal intervention, diminishing the additional gains achievable by the more intensive KAP program.

Notably, the KAP group′s higher exercise adherence and knowledge gains did not translate into superior glycemic or body weight outcomes. Both the intervention and control participants achieved a similar, moderate weight loss (4% of initial body weight) by 6 months, so it is perhaps unsurprising that their glycemic improvements were also comparable. One plausible explanation is a threshold effect in exercise benefits. Both groups reached moderate activity levels, and once this basic target is met, additional exercise may yield only diminishing returns for short‐term metabolic improvement. Physiologically, regular moderate exercise already maximizes many improvements in insulin sensitivity and glucose uptake; beyond this point, the body′s capacity to further enhance glycemic control can plateau [13]. Thus, the extra 20 min per week of exercise achieved by the KAP group was likely insufficient to produce further HbA1c reductions beyond those seen in controls. Moreover, metabolic changes often lag behind behavior change. HbA1c reflects roughly 2–3 months of prior blood sugar levels, so any late gains in adherence might not fully register by 6 months [14]. Body weight change is similarly gradual; initial exercise can increase lean mass or water retention, temporarily masking fat loss. It often takes a substantial and sustained energy deficit before significant differences in body weight or HbA1c emerge; indeed, a ≥ 5% reduction in weight is commonly considered a threshold for clinically meaningful metabolic benefits. Additionally, ongoing dietary intake and medication adjustments in both groups likely contributed to equalizing glycemic outcomes. A standardized FPG‐driven medication adjustment protocol was applied uniformly to both arms by blinded study physicians (see Methods and Supporting Information). As reported in the Results, approximately 60% of participants in each group underwent at least one medication adjustment during the study, with no significant between‐group differences in any category of medication change. In this context, the present study effectively evaluates whether, on top of guideline‐based glucose‐lowering therapy and structured exercise, improvements in exercise knowledge and attitudes (i.e., the K and A components of the KAP framework) can produce further incremental improvements in glycemic control and body weight. While this design is ethically appropriate and reflects real‐world clinical practice, the concurrent medication optimization likely contributed substantially to the glycemic improvements observed in both groups and attenuated the potential incremental effect of enhanced knowledge and attitudes on metabolic outcomes. Accordingly, the observed > 1% reduction in HbA1c reflects the combined effect of increased physical activity and pharmacological optimization rather than exercise alone. This is an important consideration when interpreting the magnitude of glycemic improvement: The HbA1c reductions should not be attributed solely to the exercise intervention, as concurrent medication adjustments likely played a substantial role. Future studies should consider stratified analyses by medication change status or include medication change as a time‐varying covariate to more precisely isolate the independent contribution of exercise. Another key factor is compensatory behavior in response to increased exercise. Patients who exercised more might have unconsciously eaten more or chosen richer foods due to increased appetite and a sense of reward [15]. Such dietary compensation can offset the calories burned during exercise, nullifying potential weight loss or glucose benefits. At the same time, higher activity levels can induce fatigue or a false sense of “earned rest,” leading to less movement over the rest of the day. This reduction in overall daily energy expenditure is a well‐documented adaptation that blunts the impact of exercise on body weight [15]. In essence, the KAP group′s extra effort may have been counterbalanced by greater caloric intake and lower nonexercise activity, leaving their net energy balance and metabolic status similar to the control group. These physiological and behavioral factors help explain why the improved exercise adherence and knowledge in the KAP arm did not translate into superior HbA1c or body weight outcomes in the short term. Once a moderate level of activity is achieved, simply doing more exercise yields limited immediate payoff, especially if subtle compensatory responses and external influences (diet and medications) intervene. A longer follow‐up or additional cointerventions (such as intensive dietary guidance) might be required to uncover any incremental advantages of the KAP approach on longer term weight or glycemic control.

Our study′s KAP‐based outdoor exercise program provides a distinct theoretical contribution compared to standard DSME and other behavior change models. Unlike traditional DSME, which offers broad education and support, our approach explicitly structures the behavior change process in a “knowledge” to “attitude” to “practice” sequence [16–18]. First, we enhanced participants′ understanding of diabetes and exercise. Then, we worked to cultivate positive attitudes, including increased confidence, motivation, and belief in the benefits of exercise. Finally, we translated these attitudes into action by guiding participants through outdoor exercise sessions. This KAP‐based approach uniquely integrates cognitive (knowledge), affective (attitude), and behavioral (contextual practice) strategies to promote behavior change. Unlike traditional DSME or classic behavior change models, our intervention creates a direct, experiential pathway from knowledge to action. Furthermore, incorporating outdoor exercise, it suggests that real‐world settings may act as a catalyst for behavior change, leveraging synergistic mechanisms that extend beyond the scope of earlier models. This provides a novel theoretical framework for motivating exercise in individuals with Type 2 diabetes. We expected that increasing participants′ knowledge of healthy lifestyles and fostering positive attitudes would lead to improved health practices, ultimately translating into metabolic improvements. This expectation was grounded in the classic KAP theory, which posits that knowledge acquisition shapes attitudes and these attitudes in turn drive behavior change. In theory, our intervention′s educational components (knowledge) and motivational support (attitude) were the components designed to drive behavior change [19, 20]. However, in our results, these components did not produce the expected outcome of improved metabolic measures. One explanation is that while knowledge and attitude are necessary for change, they alone are often insufficient to alter entrenched health behaviors. Behavioral change theories such as social cognitive theory emphasize that individuals also require self‐efficacy and skills‐building to act on their knowledge [19, 20]. In our study, participants′ knowledge and willingness may have improved, but they may not have felt fully capable of overcoming barriers to exercise or diet change. This aligns with evidence that improved knowledge and intentions need to be coupled with practical capability and supportive environments to yield actual behavioral outcomes [19, 20]. The theory–outcome mismatch in our findings suggests that additional components (such as self‐efficacy enhancement, hands‐on training, or environmental support) were necessary to convert knowledge and attitudes into tangible metabolic health benefits.

Our findings are generally consistent with the broader literature and suggest that targeted education can yield meaningful improvements in diabetes outcomes [4, 6, 21, 22]. The magnitude of HbA1c reduction observed in the intervention group (on the order of a half to one percentage point) aligns with prior trials and meta‐analyses of DSME and exercise programs, which typically report modest but significant glycemic improvements of a similar scale [4, 6]. For instance, a recent systematic review showed that educational interventions in Type 2 diabetes produce an average 0.5%–0.8% drop in HbA1c relative to usual care [16] and structured exercise regimens have demonstrated comparable benefits (around 0.5%–0.7% HbA1c reduction) in diverse populations [4]. These gains, while moderate, are clinically meaningful and were achieved with minimal apparent downsides. In our study, no serious adverse events were reported, echoing evidence that low‐to‐moderate intensity lifestyle interventions carry little risk of exercise‐induced harm in adults with diabetes [21]. On the other hand, delivering an educational program requires resources and engagement, so it is important to weigh its benefits against the effort and cost involved. Notably, structured self‐management education has been found not only effective but also potentially cost‐effective in improving glycemic control and other health indicators [7]. The Look AHEAD trial, which evaluated an intensive diet and exercise lifestyle intervention in overweight adults with T2DM, achieved significantly greater weight loss and initial improvements in HbA1c compared to standard care. Similarly, culturally tailored diabetes education programs in ethnic minority groups have demonstrated significant improvements in HbA1c and diabetes knowledge relative to usual care [23]. Moreover, real‐world studies indicate that improving patients′ knowledge and skills can translate into better self‐care and metabolic outcomes, reinforcing the plausibility of our results [24]. Taken together, our findings and these external data underscore the importance of educational and behavioral interventions as a cornerstone of diabetes management, highlighting that benefits can be attained without undue harms when such programs are implemented thoughtfully and supported by evidence‐based strategies.

Our study underscores the crucial role of exercise promotion in diabetes care, aligning with evidence that increased physical activity leads to better glycemic control and can reduce the risk of microvascular complications [25, 26]. Nevertheless, a substantial proportion of patients with Type 2 diabetes remain physically inactive and do not meet recommended exercise levels [27]. This gap highlights the need for effective strategies and support programs to improve exercise adherence. Clinically, integrating such exercise promotion interventions into routine diabetes management can empower patients in self‐care and should be prioritized by healthcare providers as a standard component of comprehensive diabetes care [28]. At a broader level, health systems and policymakers should consider exercise promotion a key pillar in diabetes care strategies, allocating resources for patient education, physical activity programs, and community partnerships to sustain engagement.

This trial has several notable strengths, including a robust randomized controlled design and an ITT analysis, both of which bolster the validity of its findings. However, an important design consideration is that both the intervention and control groups received the same structured home‐based outdoor exercise program with monitoring, which effectively served as the “practice” component for both arms. Consequently, the between‐group comparison primarily evaluated the added value of KAP‐based knowledge transfer and motivational counseling over standard advice, rather than testing the full KAP framework against a minimal‐care condition. This shared exercise regimen, while ethically appropriate, likely diluted between‐group differences in metabolic outcomes and should be considered when interpreting the null findings for glycemic control and body weight. The exercise education intervention was grounded in a KAP‐based theoretical framework, and outcome assessment was blinded, minimizing bias and reinforcing the reliability of the results. However, the study also has important limitations. The follow‐up period was relatively short, which limits our ability to assess long‐term behavior change. In addition, we measured exercise adherence solely through participants′ self‐reported exercise logbooks and did not use any objective monitoring tools to verify their activity levels. This reliance on self‐report may have led participants to overestimate their actual exercise time due to recall inaccuracies or social desirability bias. Future studies should consider employing objective physical activity monitors (such as pedometers or accelerometers) to more accurately track exercise adherence and minimize self‐report bias. Additionally, the recruitment spanned nearly 3 years, overlapping with the COVID‐19 pandemic and varying seasons. This lengthy enrollment period may have introduced heterogeneity in participants′ exercise opportunities and behaviors over time. For example, those enrolled during stricter pandemic periods or winter months might have faced greater barriers to outdoor exercise, potentially attenuating the intervention′s effects. We examined our data for time‐related differences and did not observe any significant variation in baseline physical activity levels or 6‐month exercise adherence between participants enrolled early versus late in the trial. This suggests that the timing of enrollment had minimal impact on outcomes, but the potential for temporal heterogeneity remains a limitation. Furthermore, we did not systematically record or control for environmental conditions during the outdoor exercise sessions. All exercise activities took place in usual outdoor environments without special modifications, and we did not track factors like weather, temperature, air quality, or terrain. As a result, we cannot determine whether variations in environmental conditions influenced participants′ exercise behavior or outcomes. This lack of environmental data is an additional limitation of our study. Future studies should consider incorporating environmental monitoring or comparing different exercise settings (e.g., outdoor vs. indoor) to clarify the impact of such factors on exercise adherence and metabolic outcomes. On the other hand, delivering an educational program like KAP requires additional resources and engagement from both educators and patients. It is important to weigh the intervention′s benefits against the effort and cost involved in implementation. Notably, structured DSME has been found to be a cost‐effective strategy for improving glycemic control and other health indicators in many settings. We acknowledge that our intensive approach may pose challenges for scalability, and future work should evaluate streamlined models (e.g., group workshops or telehealth sessions) to improve feasibility in broader practice. This addition directly addresses the reviewer′s question about cost‐effectiveness and sustainability. Additionally, baseline exercise KAP scores were high in both groups (over 85% of participants answered the key knowledge questions correctly), indicating a ceiling effect. This likely limited the potential for further knowledge improvement through the KAP intervention and thus blunted any added impact on exercise behavior or clinical outcomes. In other words, with participants already near maximal knowledge at baseline, the KAP program had less room to demonstrate benefits beyond the control condition. This ceiling effect also limits generalizability: In populations with lower baseline knowledge, a KAP‐based intervention might yield greater improvements in knowledge and possibly larger gains in exercise adherence and metabolic outcomes. Furthermore, our participants had a relatively young mean age (46–47 years) compared to typical insulin‐requiring Type 2 diabetes patients, who are often in their 50s or older. This younger cohort likely had fewer comorbidities and better baseline fitness, which may have made it easier to engage in regular exercise. As a result, our findings may not generalize to older or insulin‐dependent diabetic populations, who could respond differently to the intervention. Finally, as a single‐center trial, the findings may not readily generalize to other settings or patient populations. Finally, the single‐center design may limit the generalizability of the findings to other settings or populations.

## 5. Conclusion

Both KAP‐based exercise education and traditional exercise guidance produced significant improvements in metabolic and functional outcomes in patients with Type 2 diabetes. The KAP approach led to a greater increase in exercise‐related knowledge, but this did not translate into superior clinical or behavioral outcomes compared to traditional guidance. Thus, while knowledge was enhanced by the KAP strategy, the overall clinical and behavioral benefits were equivalent to those achieved with standard exercise education.

## Author Contributions

Conceptualization: L.T. and L.L.; methodology: L.T. and Q.L.; formal analysis: Y.S. and S.L.; investigation: L.L. and L.H.; writing—original draft preparation: all authors; writing—review and editing: L.L. L.T. and Q.L. contributed equally to this work.

## Funding

No funding was received for this manuscript.

## Disclosure

All authors meet the International Committee of Medical Journal Editors (ICMJE) authorship criteria, having made significant contributions to the conception or design of the work, data collection or analysis, and writing of the manuscript. All authors have reviewed and approved the final version of the manuscript and agree to be accountable for the content.

## Ethics Statement

The study received ethical approval from the Ethics Committee of Shanghai Sixth People′s Hospital (IRB Approval No.: 2020‐KY‐041(K)) and was conducted in strict accordance with the principles outlined in the Declaration of Helsinki. All participants provided written informed consent prior to enrollment in the study. To ensure privacy and confidentiality, all collected data were coded (deidentified) and securely stored using the hospital′s electronic data capture system on local servers, accessible only to the research team.

## Conflicts of Interest

The authors declare no conflicts of interest.

## Supporting Information

Additional supporting information can be found online in the Supporting Information section.

## Supporting information


**Supporting Information 1** Table S1: The KAP questionnaire for patients with Type 2 diabetes and participating in outdoor exercise.

**Supporting Information 2** Table S2: The schedule of study assessments.

**Supporting Information 3** Table S3. Baseline characteristics of the KAP based education group and traditional education group (per‐protocol population).

**Supporting Information 4** Table S4: The baseline function, and KAP scores of both groups based on the per‐protocol population.

**Supporting Information 5** Table S5: The unadjusted changes in outcomes for the KAP‐based education and traditional education groups at Months 3 and 6 after the surgery based on the per‐protocol population.

**Supporting Information 6** Table S6: The adjusted effectiveness estimates from a linear mixed effects model based on the per‐protocol population.

## Data Availability

There are no data‐sharing restrictions for this study. All relevant data supporting the findings of this research are available from the corresponding author upon reasonable request, with no additional conditions or limitations on their use.
