# Supplementary material for: Knowledge–Attitude–Practice‐Based Outdoor Exercise Education for Patients With Type 2 Diabetes: A Randomized Controlled Trial
Source: J Diabetes Res. 2026 Jun 29;2026:4523789. doi: 10.1155/jdr/4523789 (PMC13312433; doi:10.1155/jdr/4523789)
Supplement: Supplementary file 1 — Supporting Information 1 Table S1: The KAP questionnaire for patients with Type 2 diabetes and participating in outdoor exercise. [file JDR-2026-4523789-s004.docx]

**Supplementary Table 1, KAP Questionnaire for patients with type 2 diabetes and participating in outdoor exercise**

| **Questions** | **Response** |
| --- | --- |
| **Knowledge Domain (11 questions, Yes/No or factual answer):** Assesses the patient’s understanding of diabetes and exercise guidelines/benefits |  |
| 1. Regular exercise can help lower blood sugar levels in patients with type 2 diabetes. | True/False |
| 1. The recommended minimum amount of moderate-intensity exercise for adults with diabetes is 150 minutes per week. | True/False |
| 1. Exercise can improve insulin sensitivity and help insulin work better in the body. | True/False |
| 1. People with diabetes should avoid exercise if their blood glucose is extremely high (for example, >250 mg/dL with ketones) or very low (<70 mg/dL). | True/False |
| 1. Besides blood sugar control, regular physical activity can reduce the risk of heart disease and high blood pressure. | True/False |
| 1. Being physically active can help in weight loss or maintaining a healthy weight. | True/False |
| 1. It is necessary to do warm-up exercises before starting your main exercise session. | Yes/No |
| 1. Wearing comfortable shoes and inspecting your feet is important for diabetic patients when exercising. | Yes/No |
| 1. If I exercise regularly, I might be able to reduce my diabetes medication dose (with my doctor’s guidance). | True/False |
| 1. Muscle-strengthening exercises (like lifting light weights or resistance band exercises) are recommended at least 2 times a week for people with diabetes. | True/False |
| 1. Skipping exercise for a week or two will not affect diabetes control. | True/False |
| **Attitude Domain (7 questions, Likert scale):** Evaluates the patient’s beliefs, motivation, and perceived importance of exercise. Each attitude item is phrased as a statement with which the patient indicates level of agreement on a 5-point Likert scale (for example: 1 = strongly disagree, 5 = strongly agree). |  |
| 1. I believe that regular exercise is an important part of managing my diabetes | 5-point Likert scale (1 = strongly disagree, 5 = strongly agree) |
| 1. I am confident that I can exercise regularly, even if I encounter obstacles (e.g., bad weather or a busy schedule) | 5-point Likert scale (1 = strongly disagree, 5 = strongly agree) |
| 1. I enjoy being physically active. | 5-point Likert scale (1 = strongly disagree, 5 = strongly agree) |
| 1. I worry that exercise might cause me to have low blood sugar or other health problems.” | negative-attitude item – reverse scored |
| 1. I feel motivated to exercise when I think about the benefits it can bring to my health. | 5-point Likert scale (1 = strongly disagree, 5 = strongly agree) |
| 1. Exercise is as important as taking medication for controlling my diabetes. | 5-point Likert scale (1 = strongly disagree, 5 = strongly agree) |
| 1. I would exercise more if I had someone to do it with or a group for support. | 5-point Likert scale (1 = strongly disagree, 5 = strongly agree) |
| **Practice Domain (7 questions, frequency or yes/no):** Assesses the patient’s self-reported exercise behaviors and routine (the “practice” of exercise). These include questions on current exercise frequency, habits, and self-care practices. |  |
| 1. On average, how many days per week do you engage in at least 30 minutes of physical activity? | Multiple choice: 0 days, 1–2 days, 3–4 days, 5 or more days |
| 1. Do you perform a warm-up before exercising and a cool-down after exercising? | Yes/No |
| 1. Do you monitor your blood glucose (or pay attention to how you feel) before or after exercise sessions? | Yes/No |
| 1. When was the last time you exercised continuously for at least 20–30 minutes? | today, within this week, last week, more than 2 weeks ago, cannot remember |
| 1. I incorporate physical activity into my daily routine (for example, taking the stairs, walking instead of driving short distances | Likert 1–5 from never to always |
| 1. If I miss planned exercise sessions, I make an effort to resume and continue afterward. | Likert 1–5 from never to always |
| 1. Do you keep a record or log of your physical activity? | Yes/No |

Scoring: Knowledge questions are mostly true/false (or yes/no) format. Each correct answer is given 1 point, and an incorrect or “don’t know” answer 0 points. Thus, the knowledge score ranges from 0 to 11 points, with higher scores indicating greater factual knowledge about exercise in diabetes.

Each attitude item is scored 5 for the most positive attitude (e.g. strongly agree with a positive statement, or strongly disagree with a negative statement), down to 1 for the most negative. We will reverse-score any negatively phrased items (such as concern about hypoglycemia – agreement indicates a barrier, so we reverse it to reflect a lower attitude score). Summing these yields an attitude score range of 7 to 35. Higher scores mean a more positive and proactive attitude towards exercise.

Practice items use a mix of yes/no and frequency scales. We will harmonize scores to a 0–1 or 1–5 scale per item. Yes = 1, No = 0 for binary questions. Frequency questions are scored so that a higher number indicates better practice. The total practice score can range, for example, 0 to 7 (if binary) or up to 35 if using Likert for all; in our case, with a combination, we will convert to a percentage score or simply sum points (e.g. yes=1, highest frequency=5, etc.). The precise scoring scheme will be defined a priori. Higher practice scores indicate healthier exercise habits.
