# Supplementary material for: Knowledge–Attitude–Practice‐Based Outdoor Exercise Education for Patients With Type 2 Diabetes: A Randomized Controlled Trial
Source: J Diabetes Res. 2026 Jun 29;2026:4523789. doi: 10.1155/jdr/4523789 (PMC13312433; doi:10.1155/jdr/4523789)
Supplement: Supplementary file 2 — Supporting Information 2 Table S2: The schedule of study assessments. [file JDR-2026-4523789-s006.docx]

**Supplementary Table 2, Schedule of Study Assessments**

| **Measure** | **Baseline (0 mo)** | **3 Months** | **6 Months** |
| --- | --- | --- | --- |
| Informed consent, demographics | ✓ (baseline only) | – | – |
| Medical history & medications | ✓ | Update | Update |
| Physical exam (weight, BMI, BP, waist, 6MWT, CST) | ✓ | ✓ | ✓ |
| HbA1c | ✓ | ✓ | ✓ |
| Fasting glucose | ✓ | ✓ | ✓ |
| KAP Questionnaire (knowledge/attitude/practice) | ✓ | ✓ | ✓ |
| Exercise diary review | – | ✓ | ✓ |
| Physical activity self-report | ✓ (baseline PA) | ✓ | ✓ |
| Adverse events check | – (prior to intervention) | ✓ | ✓ |
| Intervention delivered | (after baseline data, randomize) | Booster education phone calls | Booster call (month 5) |
| Standard care/brief advice (control) | ✓ (at baseline) | Standard care only | Standard care only |
| QoL survey (exploratory) | ✓ | – | ✓ |

Note: “✓” indicates the assessment is performed at that timepoint. “Update” means any changes from baseline are recorded.

6MWT: 6-minute walk test distance (m); CST: Chair-stand test (in 30 sec); QoL: Quality of life.
