# Supplementary material for: Knowledge–Attitude–Practice‐Based Outdoor Exercise Education for Patients With Type 2 Diabetes: A Randomized Controlled Trial
Source: J Diabetes Res. 2026 Jun 29;2026:4523789. doi: 10.1155/jdr/4523789 (PMC13312433; doi:10.1155/jdr/4523789)
Supplement: Supplementary file 3 — Supporting Information 3 Table S3. Baseline characteristics of the KAP based education group and traditional education group (per‐protocol population). [file JDR-2026-4523789-s003.docx]

**Supplementary Table 3, Baseline characteristics of the KAP based education group and traditional education group (per-protocol population)**

| **PP** | **Characteristic** | **KAP group (N=97)** | **Control group (N=98)** | **P value** |
| --- | --- | --- | --- | --- |
| **Baseline characteristics** | **Male Gender (no.[%])** | 34 (35.05) | 35 (35.71) | 0.923 |
|  | **Age (yrs)** | 47 (10.92) | 46 (10.39) | 0.811 |
|  | **BMI (kg/m²)** | 29 (1.35) | 29 (1.51) | 0.311 |
|  | **Education Level (no.[%])** |  |  |  |
|  | below high school | 75 (77.32) | 65 (66.33) | 0.088 |
|  | ≥high school | 22 (22.68) | 33 (33.67) |  |
|  | **Insurance Type (no.[%])** |  |  |  |
|  | Government | 65 (67.01) | 54 (55.10) | 0.180 |
|  | Commercial | 18 (18.56) | 21 (21.43) |  |
|  | Self-financed | 14 (14.43) | 23 (23.47) |  |
|  | **Type of work (no.[%])** |  |  |  |
|  | Labor | 58 (59.79) | 63 (64.29) | 0.518 |
|  | Non-labor | 39 (40.21) | 35 (35.71) |  |
| **Medical History** | **Duration of diabetes (years)** | 9 (3.07) | 9 (3.17) | 0.668 |
|  | **Insulin use (no.[%])** | 78 (80.41) | 75 (76.53) | 0.510 |
|  | **Oral antidiabetic medications (no.[%])** | 81 (83.51) | 76 (77.55) | 0.294 |
|  | Metformin | 71 (87.7) | 66 (86.8) | 0.822 |
|  | Sulfonylureas | 18 (22.2) | 16 (21.1) | 0.887 |
|  | DPP-4 inhibitors | 32 (39.5) | 28 (36.8) | 0.822 |
|  | Thiazolidinediones | 24 (29.6) | 21 (27.6) | 0.822 |
|  | α-glucosidase inhibitors | 58 (71.6) | 55 (72.4) | 0.887 |
|  | **Hypertension (no.[%])** | 7 (7.22) | 11 (11.22) | 0.334 |
|  | **Cardiovascular diseases (no.[%])** | 8 (8.25) | 12 (12.24) | 0.358 |
|  | **Atrial fibrillation (no.[%])** | 2 (2.06) | 3 (3.06) | 0.659 |
|  | **COPD (no.[%])** | 2 (2.06) | 4 (4.08) | 0.414 |
|  | **Arthritis/connective tissue disease (no.[%])** | 9 (9.28) | 12 (12.24) | 0.504 |
|  | **Dyslipidemia (no.[%])** | 8 (8.25) | 9 (8.18) | 0.817 |
|  | **Hypothyroidism (no.[%])** | 2 (2.06) | 3 (3.06) | 0.659 |

PP: per-protocol population; KAP: knowledge, attitude, and practice; BMI: body mass index; COPD: Chronic Obstructive Pulmonary Disease

Values were reported as mean (standard deviation) for age and BMI, others were reported as number (percentage).
