# Supplementary material for: Knowledge–Attitude–Practice‐Based Outdoor Exercise Education for Patients With Type 2 Diabetes: A Randomized Controlled Trial
Source: J Diabetes Res. 2026 Jun 29;2026:4523789. doi: 10.1155/jdr/4523789 (PMC13312433; doi:10.1155/jdr/4523789)
Supplement: Supplementary file 4 — Supporting Information 4 Table S4: The baseline function, and KAP scores of both groups based on the per‐protocol population. [file JDR-2026-4523789-s001.docx]

**Supplementary Table 4, Baseline function and KAP scores of both groups (per-protocol population)**

| **PP** | **Characteristic** | **KAP group (N=97)** | **Control group (N=98)** | **P value** |
| --- | --- | --- | --- | --- |
| **Function outcome** | Weight (kg) | 90 (6.09) | 90 (6.86) | 0.657 |
|  | Waist circumference (cm) | 87 (8.67) | 86 (8.06) | 0.213 |
|  | Resting systolic blood pressure (mmHg) | 129 (10.63) | 128 (10.52) | 0.822 |
|  | Resting diastolic blood pressure (mmHg) | 90 (5.44) | 88 (5.61) | 0.081 |
|  | Resting heart rate (bpm) | 74 (9.61) | 75 (9.18) | 0.239 |
| **Laboratory Test Outcomes** | HbA1c (%) | 8 (0.47) | 8 (0.48) | 0.427 |
|  | Fasting plasma glucose (mg/dL) | 113 (18.17) | 115 (16.92) | 0.561 |
|  | Fasting insulin (µIU/mL) | 7 (1.13) | 7 (1.17) | 0.713 |
|  | Triglycerides (mg/dL) | 165 (30.80) | 160 (32.78) | 0.246 |
| **Functional Exercise Capacity** | 6-minute walk test distance (m) | 440 (79.08) | 454 (86.80) | 0.239 |
|  | Chair-stand test (in 30 sec) | 14 (2.80) | 14 (2.68) | 0.332 |
| **Patient-Reported Outcome Measures** | SF-36 physical functioning (score) | 63 (5.25) | 62 (5.62) | 0.669 |
|  | SF-36 Role-physical (score) | 63 (12.56) | 61 (12.52) | 0.523 |
|  | SF-36 Bodily pain (score) | 63 (7.74) | 64 (7.20) | 0.622 |
|  | SF-36 Physical Health (score) | 63 (5.26) | 64 (4.68) | 0.551 |
|  | SF-36 Vitality (score) | 62 (8.10) | 64 (6.62) | 0.130 |
|  | SF-36 Social Functioning (score) | 60 (6.70) | 60 (7.27) | 0.951 |
|  | SF-36 Role-Emotional (score) | 59 (19.33) | 66 (18.93) | 0.012 |
|  | SF-36 Mental Health (score) | 63 (5.95) | 62 (5.92) | 0.106 |
|  | Physical Component Summary (score) | 63 (4.20) | 63 (4.07) | 0.824 |
|  | Mental Component Summary (score) | 61 (5.36) | 63 (5.48) | 0.021 |
| **KAP Knowledge domain (True; False)** | Regular exercise can help lower blood sugar levels in patients with type 2 diabetes.” (no.[%]) |  |  | 0.035 |
|  | True | 85 (87.63) | 94 (95.92) |  |
|  | False | 12 (12.37) | 4 (4.08) |  |
|  | The recommended minimum amount of moderate-intensity exercise for adults with diabetes is 150 minutes per week.” (no.[%]) |  |  | 0.197 |
|  | True | 86 (88.66) | 92 (93.88) |  |
|  | False | 11 (11.34) | 6 (6.12) |  |
|  | Exercise can improve insulin sensitivity and help insulin work better in the body.” (no.[%]) |  |  | 0.654 |
|  | True | 88 (90.72) | 87 (88.78) |  |
|  | False | 9 (9.28) | 11 (11.22) |  |
|  | People with diabetes should avoid exercise if their blood glucose is extremely high (for example, >250 mg/dL with ketones) or very low (<70 mg/dL).” (assesses safety knowledge) (no.[%]) |  |  | 0.987 |
|  | True | 92 (94.85) | 93 (94.90) |  |
|  | False | 5 (5.15) | 5 (5.10) |  |
|  | Besides blood sugar control, regular physical activity can reduce the risk of heart disease and high blood pressure.” (no.[%]) |  |  | 0.454 |
|  | True | 86 (88.66) | 90 (91.84) |  |
|  | False | 11 (11.34) | 8 (8.16) |  |
|  | Being physically active can help in weight loss or maintaining a healthy weight.” (no.[%]) |  |  | 0.114 |
|  | True | 82 (84.54) | 90 (91.84) |  |
|  | False | 15 (15.46) | 8 (8.16) |  |
|  | It is necessary to do warm-up exercises before starting your main exercise session.” (no.[%]) |  |  | 0.144 |
|  | Yes | 91 (93.81) | 86 (87.76) |  |
|  | No | 6 (6.19) | 12 (12.24) |  |
|  | Wearing comfortable shoes and inspecting your feet is important for diabetic patients when exercising.” (no.[%]) |  |  | 0.982 |
|  | Yes | 88 (90.72) | 89 (90.82) |  |
|  | No | 9 (9.28) | 9 (9.18) |  |
|  | If I exercise regularly, I might be able to reduce my diabetes medication dose (with my doctor’s guidance).” (no.[%]) |  |  | 0.189 |
|  | True | 75 (77.32) | 83 (84.69) |  |
|  | False | 22 (22.68) | 15 (15.31) |  |
|  | Muscle-strengthening exercises (like lifting light weights or resistance band exercises) are recommended at least 2 times a week for people with diabetes.” (no.[%]) |  |  | 0.772 |
|  | True | 89 (91.75) | 91 (92.86) |  |
|  | False | 8 (8.25) | 7 (7.14) |  |
|  | Skipping exercise for a week or two will not affect diabetes control.” (no.[%]) |  |  | 0.463 |
|  | True | 23 (23.71) | 19 (19.39) |  |
|  | False | 74 (76.29) | 79 (80.61) |  |
| **KAP Attitude domain (Likert scale, 5 for the most positive attitude)** | I believe that regular exercise is an important part of managing my diabetes. (no.[%]) |  |  | 0.646 |
|  | Strongly Disagree | 0 (0.00) | 0 (0.00) |  |
|  | Disagree | 0 (0.00) | 0 (0.00) |  |
|  | Neutral | 13 (13.40) | 15 (15.31) |  |
|  | Agree | 41 (42.27) | 46 (46.94) |  |
|  | Strongly agree | 43 (44.33) | 37 (37.76) |  |
|  | I am confident that I can exercise regularly, even if I encounter obstacles (e.g., bad weather or a busy schedule) (no.[%]) |  |  | 0.136 |
|  | Strongly Disagree | 0 (0.00) | 0 (0.00) |  |
|  | Disagree | 0 (0.00) | 0 (0.00) |  |
|  | Neutral | 10 (10.31) | 17 (17.35) |  |
|  | Agree | 40 (41.24) | 46 (46.94) |  |
|  | Strongly agree | 47 (48.45) | 35 (35.71) |  |
|  | I enjoy being physically active. (no.[%]) |  |  | 0.759 |
|  | Strongly Disagree | 0 (0.00) | 0 (0.00) |  |
|  | Disagree | 0 (0.00) | 0 (0.00) |  |
|  | Neutral | 22 (22.68) | 19 (19.39) |  |
|  | Agree | 36 (37.11) | 41 (41.84) |  |
|  | Strongly agree | 39 (40.21) | 38 (38.78) |  |
|  | I worry that exercise might cause me to have low blood sugar or other health problems.” (no.[%]) |  |  | 0.039 |
|  | Strongly Disagree | 0 (0.00) | 0 (0.00) |  |
|  | Disagree | 0 (0.00) | 0 (0.00) |  |
|  | Neutral | 28 (28.87) | 14 (14.29) |  |
|  | Agree | 32 (32.99) | 43 (43.88) |  |
|  | Strongly agree | 37 (38.14) | 41 (41.84) |  |
|  | I feel motivated to exercise when I think about the benefits it can bring to my health. (no.[%]) |  |  | 0.772 |
|  | Strongly Disagree | 0 (0.00) | 0 (0.00) |  |
|  | Disagree | 0 (0.00) | 0 (0.00) |  |
|  | Neutral | 26 (26.80) | 23 (23.47) |  |
|  | Agree | 36 (37.11) | 41 (41.84) |  |
|  | Strongly agree | 35 (36.08) | 34 (34.69) |  |
|  | Exercise is as important as taking medication for controlling my diabetes. (no.[%]) |  |  | 0.324 |
|  | Strongly Disagree | 0 (0.00) | 0 (0.00) |  |
|  | Disagree | 0 (0.00) | 0 (0.00) |  |
|  | Neutral | 24 (24.74) | 16 (16.33) |  |
|  | Agree | 36 (37.11) | 38 (38.78) |  |
|  | Strongly agree | 37 (38.14) | 44 (44.90) |  |
|  | I would exercise more if I had someone to do it with or a group for support. (no.[%]) |  |  | 0.213 |
|  | Strongly Disagree | 0 (0.00) | 0 (0.00) |  |
|  | Disagree | 0 (0.00) | 0 (0.00) |  |
|  | Neutral | 16 (16.49) | 25 (25.51) |  |
|  | Agree | 41 (42.27) | 32 (32.65) |  |
|  | Strongly agree | 40 (41.24) | 41 (41.84) |  |
| **KAP practice domain (frequency or yes/no)** | On average, how many days per week do you engage in at least 30 minutes of physical activity?” (Multiple choice: 0 days, 1–2 days, 3–4 days, 5 or more days). This will be scored (e.g. 0=0, 1, 2, 3, 4, 5 points for 5+ days) (no.[%]) |  |  | <0.001 |
|  | 0 | 11 (11.34) | 12 (12.24) |  |
|  | 1 | 27 (27.84) | 22 (22.45) |  |
|  | 2 | 10 (10.31) | 18 (18.37) |  |
|  | 3 | 24 (24.74) | 20 (20.41) |  |
|  | 4 | 20 (20.62) | 22 (22.45) |  |
|  | 5 | 5 (5.15) | 4 (4.08) |  |
|  | Do you perform a warm-up before exercising and a cool-down after exercising?” (no.[%]) |  |  | 0.426 |
|  | Yes | 39 (40.21) | 34 (34.69) |  |
|  | No | 58 (59.79) | 64 (65.31) |  |
|  | Do you monitor your blood glucose (or pay attention to how you feel) before or after exercise sessions?” (no.[%]) |  |  | 0.242 |
|  | Yes | 69 (71.13) | 62 (63.27) |  |
|  | No | 28 (28.87) | 36 (36.73) |  |
|  | When was the last time you exercised continuously for at least 20–30 minutes?” (Options: today, within this week, last week, more than 2 weeks ago, cannot remember – to gauge recency of exercise habit) (no.[%]) |  |  | <0.001 |
|  | Cannot remember | 7 (7.22) | 5 (5.10) |  |
|  | More than 2 weeks ago | 4 (4.12) | 7 (7.14) |  |
|  | Last week | 27 (27.84) | 22 (22.45) |  |
|  | Within this week | 37 (38.14) | 29 (29.59) |  |
|  | Today | 22 (22.68) | 35 (35.71) |  |
|  | I incorporate physical activity into my daily routine (for example, taking the stairs, walking instead of driving short distances).” (Likert 1–5 from never to always) (no.[%]) |  |  | <0.001 |
|  | Never | 11 (11.34) | 12 (12.24) |  |
|  | Rarely | 23 (23.71) | 19 (19.39) |  |
|  | Sometimes | 17 (17.53) | 17 (17.35) |  |
|  | Often | 21 (21.65) | 25 (25.51) |  |
|  | Always | 25 (25.77) | 25 (25.51) |  |
|  | If I miss planned exercise sessions, I make an effort to resume and continue afterward.” (Likert 1–5 from never to always) (no.[%]) |  |  | <0.001 |
|  | Never | 0 (0.00) | 0 (0.00) |  |
|  | Rarely | 23 (17.16) | 17 (12.41) |  |
|  | Sometimes | 14 (10.45) | 15 (10.95) |  |
|  | Often | 23 (17.16) | 27 (19.71) |  |
|  | Always | 37 (27.61) | 39 (28.47) |  |
|  | Do you keep a record or log of your physical activity? (no.[%]) |  |  | 0.353 |
|  | Yes | 46 (47.42) | 40 (40.82) |  |
|  | No | 51 (52.58) | 58 (59.18) |  |

Values were reported as mean (standard deviation) for Physical Examination Outcome, Laboratory Test Outcomes, Functional Exercise Capacity and Patient-Reported Outcome Measures. Values for all KAP domains were reported as number (percentage)
