# Supplementary material for: Knowledge–Attitude–Practice‐Based Outdoor Exercise Education for Patients With Type 2 Diabetes: A Randomized Controlled Trial
Source: J Diabetes Res. 2026 Jun 29;2026:4523789. doi: 10.1155/jdr/4523789 (PMC13312433; doi:10.1155/jdr/4523789)
Supplement: Supplementary file 5 — Supporting Information 5 Table S5: The unadjusted changes in outcomes for the KAP‐based education and traditional education groups at Months 3 and 6 after the surgery based on the per‐protocol population. [file JDR-2026-4523789-s002.docx]

**Supplementary Table 5, Unadjusted changes in outcomes for the KAP based education and traditional education groups at months 3, and 6 after the surgery (per-protocol population)**

| **Per-protocol population** | **3 months** | | |  | **6 months** | | |
| --- | --- | --- | --- | --- | --- | --- | --- |
|  | **KAP group (N=97)** | **Control group (N=98)** | **P value** |  | **KAP group (N=97)** | **Control group (N=98)** | **P value** |
| **Physical Examination Outcome** |  |  |  |  |  |  |  |
| Weight (kg) | 89.59 (6.04) | 89.13 (6.73) | 0.620 |  | 86.48 (5.86) | 86.07 (6.51) | 0.646 |
| Total weight loss (kg) | 0.56 (0.31) | 0.60 (0.33) | 0.360 |  | 3.67 (0.74) | 3.67 (0.87) | 0.967 |
| BMI (kg/m2) | -0.20 (0.05) | -0.20 (0.05) | 0.763 |  | -1.20 (0.20) | -1.19 (0.23) | 0.579 |
| Waist circumference (cm) | -3.06 (0.58) | -3.01 (0.51) | 0.529 |  | -6.06 (1.21) | -3.01 (0.51) | 0.658 |
| Resting systolic blood pressure (mmHg) | -3.10 (0.53) | -2.98 (0.58) | 0.203 |  | -6.05 (0.83) | -2.98 (0.58) | 0.744 |
| Resting diastolic blood pressure (mmHg) | -1.98 (0.55) | -2.01 (0.47) | 0.715 |  | -3.95 (0.70) | -2.01 (0.47) | 0.697 |
| Resting heart rate (bpm) | -1.00 (0.38) | -1.07 (0.46) | 0.355 |  | -2.53 (0.64) | -1.07 (0.46) | 0.502 |
| **Laboratory Test Outcomes** |  |  |  |  |  |  |  |
| HbA1c (%) | -0.75 (0.17) | -0.76 (0.16) | 0.743 |  | -1.28 (0.18) | -0.76 (0.16) | 0.364 |
| Fasting plasma glucose (mg/dL) | -0.33 (0.08) | -0.34 (0.06) | 0.544 |  | -0.50 (0.08) | -0.50 (0.07) | 0.677 |
| Fasting insulin (µIU/mL) | -0.50 (0.10) | -0.51 (0.10) | 0.449 |  | -0.99 (0.15) | -0.51 (0.10) | 0.283 |
| Triglycerides (mg/dL) | 0.00 (0.00) | -0.01 (0.00) | 0.589 |  | -0.01 (0.00) | -0.01 (0.00) | 0.380 |
| **Functional Exercise Capacity** |  |  |  |  |  |  |  |
| 6-minute walk test distance (m) | 30.14 (4.24) | 30.05 (4.33) | 0.639 |  | 50.32 (5.43) | 30.05 (4.33) | 0.873 |
| Chair-stand test (in 30 sec) | 1.93 (0.41) | 2.03 (0.46) | 0.071 |  | 2.92 (0.53) | 2.03 (0.46) | 0.014 |
| **Patient-Reported Outcome Measures** |  |  |  |  |  |  |  |
| SF-36 physical functioning | 5.41 (4.25) | 5.39 (4.38) | 0.940 |  | 12.73 (5.05) | 5.39 (4.38) | 0.707 |
| SF-36 Role-physical | 13.66 (12.51) | 11.03 (12.47) | 0.175 |  | 23.45 (15.23) | 11.03 (12.47) | 0.914 |
| SF-36 Bodily pain | 6.27 (6.25) | 6.03 (6.19) | 0.781 |  | 12.44 (9.89) | 6.03 (6.19) | 0.879 |
| SF-36 Physical Health | 5.72 (4.21) | 4.80 (4.22) | 0.175 |  | 13.56 (4.62) | 4.80 (4.22) | 0.237 |
| SF-36 Vitality | 4.02 (3.79) | 5.78 (3.71) | 0.001 |  | 11.65 (4.61) | 5.78 (3.71) | 0.004 |
| SF-36 Social Functioning | 5.02 (5.54) | 5.86 (5.56) | 0.350 |  | 10.37 (8.09) | 5.86 (5.56) | 0.996 |
| SF-36 Role-Emotional | 17.20 (16.76) | 10.41 (20.98) | 0.015 |  | 25.75 (19.59) | 10.41 (20.98) | 0.025 |
| SF-36 Mental Health | 4.00 (3.11) | 3.76 (3.27) | 0.656 |  | 8.29 (4.17) | 3.76 (3.27) | 0.147 |
| **KAP scores** |  |  |  |  |  |  |  |
| Scores in Knowledge domain | 0.19 (0.89) | -0.05 (0.85) | 0.049 |  | 0.23 (0.94) | -0.05 (0.85) | 0.041 |
| Scores in Attitude domain | 2.26 (1.70) | 2.27 (1.58) | 0.871 |  | 2.68 (1.72) | 2.27 (1.58) | 0.251 |
| Scores in Practice domain | 5.65 (2.50) | 5.70 (2.55) | 0.872 |  | 6.73 (3.18) | 5.70 (2.55) | 0.608 |

BMI: body mass index; KAP: knowledge, Attitude and Practice

Values represent the mean change from baseline for each group, reported as mean (standard deviation), with P values comparing between-group differences at each follow-up point. All outcome measures were unadjusted.
