# Supplementary material for: Knowledge–Attitude–Practice‐Based Outdoor Exercise Education for Patients With Type 2 Diabetes: A Randomized Controlled Trial
Source: J Diabetes Res. 2026 Jun 29;2026:4523789. doi: 10.1155/jdr/4523789 (PMC13312433; doi:10.1155/jdr/4523789)
Supplement: Supplementary file 6 — Supporting Information 6 Table S6: The adjusted effectiveness estimates from a linear mixed effects model based on the per‐protocol population. [file JDR-2026-4523789-s005.docx]

**Supplementary Table 6, Adjusted effectiveness estimates from linear mixed effects models (per-protocol population)**

| **Per-protocol population** | **3 months** | | |  | **6 months** | | |
| --- | --- | --- | --- | --- | --- | --- | --- |
|  | **Coefficient** | **95% CI** | **P value** |  | **Coefficient** | **95% CI** | **P value** |
| **Physical Examination Outcome** |  |  |  |  |  |  |  |
| Weight (kg) | 0.017 | (-0.022, 0.055) | 0.398 |  | 0.010 | (-0.078, 0.098) | 0.823 |
| Total weight loss (kg) | -0.017 | (-0.055, 0.022) | 0.398 |  | -0.010 | (-0.098, 0.078) | 0.823 |
| BMI (kg/m2) | 0.000 | (-0.01, 0.01) | 0.925 |  | -0.004 | (-0.03, 0.02) | 0.732 |
| Waist circumference (cm) | 1.056 | (-1.23, 3.35) | 0.366 |  | 1.045 | (-1.25, 3.34) | 0.372 |
| Resting systolic blood pressure (mmHg) | -0.538 | (-3.26, 2.19) | 0.698 |  | -0.524 | (-3.25, 2.21) | 0.707 |
| Resting diastolic blood pressure (mmHg) | 0.919 | (-0.44, 2.28) | 0.187 |  | 0.932 | (-0.43, 2.30) | 0.181 |
| Resting heart rate (bpm) | -1.687 | (-4.33, 0.95) | 0.211 |  | -1.716 | (-4.36, 0.93) | 0.204 |
| **Laboratory Test Outcomes** |  |  |  |  |  |  |  |
| HbA1c (%) | -0.051 | (-0.18, 0.08) | 0.457 |  | -0.058 | (-0.19, 0.08) | 0.402 |
| Fasting plasma glucose (mg/dL) | -0.061 | (-0.260, 0.139) | 0.551 |  | -0.066 | (-0.263, 0.130) | 0.509 |
| Fasting insulin (µIU/mL) | -0.074 | (-0.24, 0.09) | 0.371 |  | -0.069 | (-0.23, 0.09) | 0.409 |
| Triglycerides (mg/dL) | 0.328 | (-0.203, 0.859) | 0.226 |  | 0.322 | (-0.208, 0.853) | 0.234 |
| **Functional Exercise Capacity** |  |  |  |  |  |  |  |
| 6-minute walk test distance (m) | -14.362 | (-37.61, 8.89) | 0.226 |  | -14.411 | (-37.73, 8.90) | 0.226 |
| Chair-stand test (in 30 sec) | 0.196 | (-0.51, 0.90) | 0.586 |  | 0.154 | (-0.55, 0.86) | 0.669 |
| **Patient-Reported Outcome Measures** |  |  |  |  |  |  |  |
| SF-36 physical functioning | 0.531 | (-1.14, 2.20) | 0.534 |  | 0.535 | (-1.25, 2.32) | 0.558 |
| SF-36 Role-physical | 2.902 | (-0.85, 6.66) | 0.130 |  | 2.591 | (-1.20, 6.38) | 0.180 |
| SF-36 Bodily pain | -0.042 | (-2.36, 2.28) | 0.971 |  | 0.027 | (-2.52, 2.58) | 0.983 |
| SF-36 General Health | -0.236 | (-1.79, 1.32) | 0.765 |  | -0.174 | (-1.81, 1.46) | 0.835 |
| SF-36 Vitality | -2.289 | (-4.53, -0.04) | 0.046 |  | -2.579 | (-4.89, -0.27) | 0.029 |
| SF-36 Social Functioning | -0.460 | (-2.38, 1.46) | 0.638 |  | -0.348 | (-2.47, 1.78) | 0.748 |
| SF-36 Role-Emotional | -4.124 | (-9.12, 0.87) | 0.106 |  | -2.983 | (-7.79, 1.83) | 0.224 |
| SF-36 Mental Health | 1.282 | (-0.47, 3.04) | 0.152 |  | 1.699 | (-0.13, 3.52) | 0.068 |
| Physical Component Summary (score) | 0.788 | (-0.50, 2.08) | 0.230 |  | 0.745 | (-0.61, 2.10) | 0.283 |
| Mental Component Summary (score) | -1.398 | (-2.90, 0.10) | 0.068 |  | -1.053 | (-2.55, 0.44) | 0.168 |
| **KAP scores** |  |  |  |  |  |  |  |
| Scores in Knowledge domain | -0.152 | (-0.39, 0.08) | 0.201 |  | -0.105 | (-0.30, 0.09) | 0.292 |
| Scores in Attitude domain | 0.303 | (-0.12, 0.72) | 0.156 |  | 0.212 | (-0.15, 0.57) | 0.246 |
| Scores in Practice domain | -0.234 | (-1.05, 0.58) | 0.574 |  | -0.148 | (-0.77, 0.47) | 0.641 |

BMI: body mass index; KAP: knowledge, Attitude and Practice

Each coefficient represents the estimated between-group difference in the change from baseline (Intervention group minus Control group) for the specified outcome at that follow-up time point. Positive coefficients indicate higher scores in the Intervention group compared to the Control group, whereas negative values indicate lower scores in the Intervention group. Each estimate is presented with its 95% confidence interval (CI) and corresponding P value. All outcome measures were adjusted for baseline values in the model.
